# An Anaerobic Microbial Community Mediates Epigenetic Native Sulfur and Carbonate Formation During Replacement of Messinian Gypsum at Monte Palco, Sicily

**DOI:** 10.1111/gbi.70015

**Published:** 2025-03-06

**Authors:** Simon E. Rouwendaal, Daniel Birgel, Marcello Natalicchio, Francesco Dela Pierre, Laetitia Guibourdenche, Thorsten Bauersachs, Giovanni Aloisi, Amanda L. Labrado, Benjamin Brunner, Jörn Peckmann

**Affiliations:** ^1^ Fachbereich Erdsystemwissenschaften Centrum für Erdsystemforschung und Nachhaltigkeit, Universität Hamburg Hamburg Germany; ^2^ Dipartimento di Scienze della Terra Università degli Studi di Torino Torino Italy; ^3^ Institut de Physique du Globe de Paris Centre National de la Rechereche Scientifique (CNRS), Université Paris Cité Paris France; ^4^ Department of Earth, Planetary, and Space Sciences University of California Los Angeles Los Angeles California USA; ^5^ Lehrstuhl für Organische Biogeochemie in Geo‐Systemen Rheinisch‐Westfälische Technische Hochschule Aachen Aachen Germany; ^6^ Department of Air‐Sea Interaction and Remote Sensing The Applied Physics Laboratory—University of Washington Seattle Washington USA; ^7^ Department of Earth, Environmental and Resource Sciences The University of Texas at El Paso El Paso Texas USA

**Keywords:** anaerobic oxidation of methane, bacterial sulfate reduction, carbonate authigenesis, gypsum replacement, lipid biomarkers, microbial sulfur cycle, native sulfur

## Abstract

The microbially mediated replacement of sulfate‐bearing evaporites by authigenic carbonate and native sulfur under anoxic conditions is poorly understood. Sulfur‐bearing carbonates from the Monte Palco ridge (Sicily) replacing Messinian gypsum were therefore studied to better characterize the involved microorganisms. The lack of (1) sedimentary bedding, (2) lamination, and (3) significant water‐column‐derived lipid biomarkers in the secondary carbonates implies replacement after gypsum deposition (epigenesis). Allochthonous clasts from the older Calcare di Base and the younger Trubi Formation within these carbonates further evidence epigenetic formation. The sulfur‐bearing carbonates are significantly ^13^C‐depleted (δ^13^C as low as −51‰), identifying methane as a major carbon source. The ^18^O‐enrichment of the carbonates (δ^18^O as high as 5.4‰) probably reflects precipitation from ^18^O‐enriched fluids transported along adjacent faults or precipitation in a closed system with very little water. Native sulfur with variable ^34^S‐enrichment (δ^34^S as high as 18.9‰), a relatively small maximum offset (12.3‰) between the sulfate source (gypsum) and native sulfur, and high δ^34^S values of carbonate‐associated sulfate (as high as 61.1‰) suggest a high conversion to native sulfur in a (semi‐)closed system, with insignificant sulfate removal. Anaerobic methanotrophic archaea (ANME) apparently affiliated with the ANME‐1 clade mediated secondary mineral formation as evidenced by the biomarker inventory, which contains abundant ^13^C‐depleted isoprenoids including *sn3*‐hydroxyarchaeol as the sole hydroxyarchaeol isomer and glycerol dibiphytanyl glycerol tetraethers (GDGTs). A series of various, tentatively identified ^13^C‐depleted non‐isoprenoidal dialkyl glycerol diethers (DAGEs), 10me‐C_16_ fatty acid, hydroxy C_16_ fatty acids, and cyclopropyl‐C_17:0ω7,8_ fatty acid agree with sulfate‐reducing bacteria participating in the anaerobic oxidation of methane. Specific conditions during gypsum replacement, unlike those at marine methane seeps, are reflected by the occurrence of ^13^C‐depleted lipids such as lycopane, 9me‐C_17_ fatty acid, and novel DAGEs. As a response to a confined environment probably characterized by high sulfate concentrations, sulfidic conditions, and elevated salinity, ANMEs and sulfate‐reducing bacteria apparently adapted their membrane compositions to cope with such stressors.

## 1 Introduction

Microbially mediated replacement of sulfate minerals (gypsum and/or anhydrite) is a biogeochemical process that affects various evaporite deposits and cap rocks in the subsurface (Feely and Kulp 1957; Davis and Kirkland 1979; Caesar et al. 2019). When water infiltrates and dissolves sulfate‐bearing evaporites, calcium and sulfate ions are released. If sufficient organic compounds (organic matter, oil, and methane) are available, anaerobic microorganisms gain energy from sulfate reduction coupled to the oxidation of such compounds. Several bacterial lineages are capable of sulfate reduction, most of which belong to *Deltaproteobacteria* (see Muyzer and Stams 2008 for review). Conversely, only a few archaea are capable of sulfate reduction, such as the thermophilic *Archaeoglobus* and possibly some methanotrophs (e.g., Mori et al. 2008; Steinsbu et al. 2010; Milucka et al. 2012). Sulfate reduction produces hydrogen sulfide and raises the alkalinity with bicarbonate production through organic carbon oxidation; consequently, it favors carbonate authigenesis (Thode et al. 1954; Sassen et al. 1988).

Authigenic carbonates commonly co‐occur with native sulfur resulting from the replacement of sulfate‐bearing evaporites. Historically, this type of native sulfur was explained by abiotic or biotic oxidation of hydrogen sulfide with molecular oxygen as the electron acceptor (Ivanov 1968; Davis and Kirkland 1979). The formation of native sulfur can occur during sedimentation or shortly after deposition, which is referred to as syngenesis (e.g., Druckman et al. 1994; Ziegenbalg et al. 2010; Lindtke et al. 2011). When the replacement of sulfate‐bearing evaporites takes place long after deposition during late diagenesis, which is referred to as epigenesis, the sulfur isotope composition of different sulfur‐bearing minerals has been found to suggest sulfide oxidation by electron acceptors other than molecular oxygen (Labrado et al. 2019). Rather, sulfate‐reducing or possibly other anaerobic microbes mediate native sulfur formation directly as a response to high salinities and/or high sulfide concentrations. Several mechanisms have been proposed by Labrado et al. (2019) to explain the anaerobic sulfur‐producing process, but it yet remains poorly constrained.

Bioepigenetic sulfur deposits used to be of great economic importance (Davis and Kirkland 1979) but are nowadays relatively redundant because of sulfur production as a byproduct from the fossil fuel industry—an importance that might revive when human society implements decarbonization. Bioepigenetic sulfur deposits are still of great scientific value as archives of past deep biosphere activity. Poor accessibility to active systems hinders in situ studies of subsurface microbial communities. Fortunately, salt diapirism and tectonic activity can move the replacement products of sulfate‐bearing evaporites, formed at depth, up to the surface. These rocks can be studied with (bio)geochemical techniques, such as lipid biomarker analysis, to decipher past microbial processes. Lipid biomarker analysis involves the analysis of lipids, a group of organic compounds that are part of the cell membranes in all domains in the tree of life. Lipid biomarkers can be fossilized in geological samples due to their insolubility in water and high stability toward degradation. Other than their source specificity, lipid biomarkers can also indicate certain environmental conditions (for reviews see Newman et al. 2016; Summons et al. 2022). Lipid biomarker analyses helped to confirm the contribution of microbial communities involved in the epigenetic formation of carbonate and native sulfur for Zechstein deposits of Germany (Peckmann et al. 1999), Middle Miocene deposits of Egypt (Aloisi et al. 2013), Messinian deposits of Sicily (Ziegenbalg et al. 2012), and Late Miocene deposits of the Lorca Basin in southeastern Spain (Rouwendaal et al. 2023). Combined with compound‐specific δ^13^C analysis, these analyses can also help determine microbial respiration processes, such as methanogenesis and anaerobic methane oxidation (e.g., Ziegenbalg et al. 2012; Rouwendaal et al. 2023). Still, the microbes involved in epigenetic carbonate and sulfur formation have never been identified directly, and previous results indicate variable environmental conditions and different microbial communities.

**FIGURE 1:**
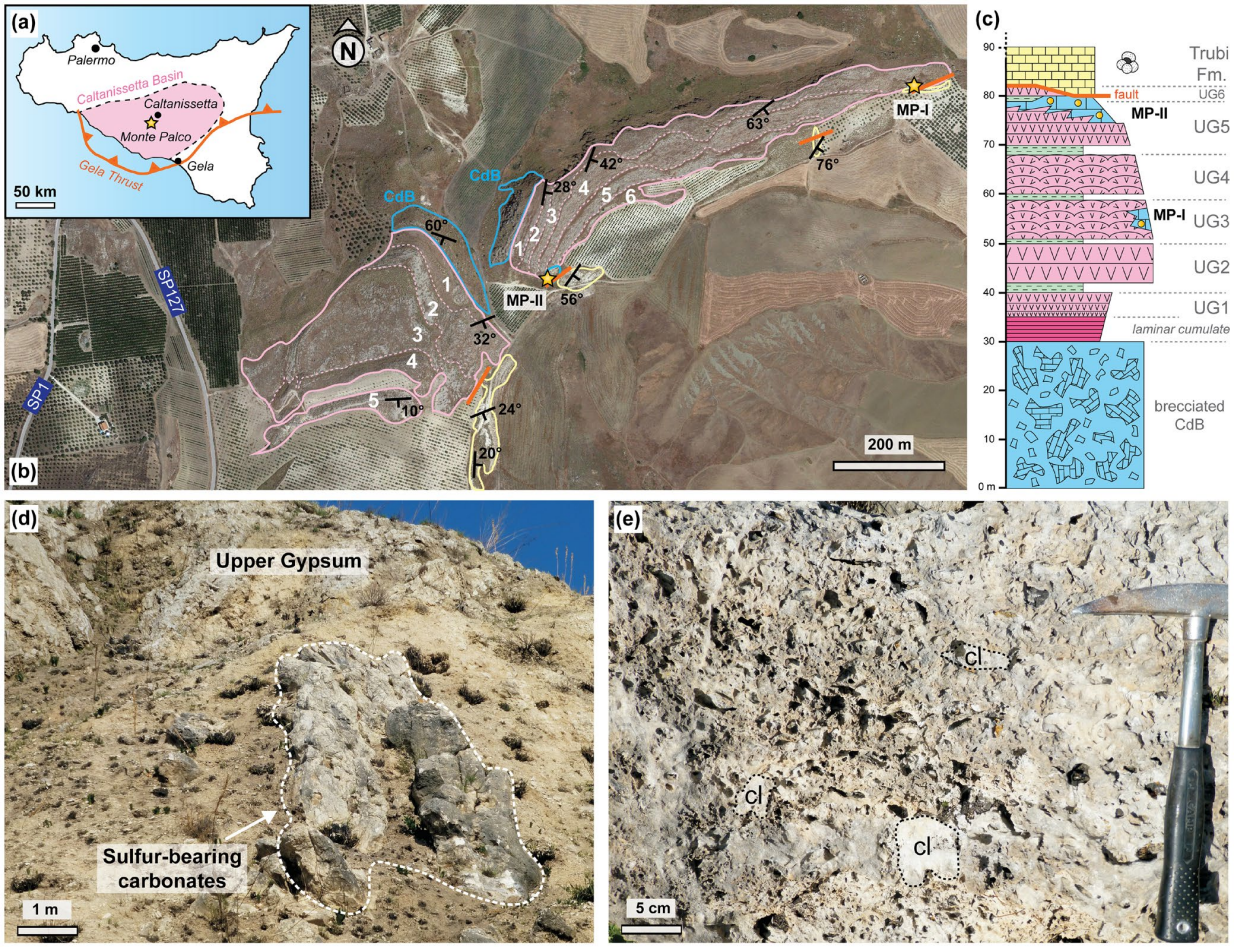
Location map of the Monte Palco section. (a) Map of Sicily highlighting the Caltanissetta Basin, the studied Monte Palco section (star) and the Gela Thrust. (b) Satellite image of the Monte Palco section with lithological units, measured strikes and dips, and the sampling sites (stars). Lithological units comprise limestones, including brecciated Calcare di Base (CdB) at the base and sulfur‐bearing carbonates (blue), Upper Gypsum (UG) cycles 1–6 (pink) and the foraminifera‐bearing Trubi Formation (yellow). Supposed fault is displayed in orange. (c) Reconstructed stratigraphic column for the Monte Palco area. The numbered UG labels display the various UG cycles. Green layers are composed of marls and pink layers of bottom‐grown gypsum. Yellow dots indicate the stratigraphic positions of the samples from the two sampling locations (“MP‐I” and “MP‐II”). Unit classification modified after Manzi et al. (2009). A detailed description of the CdB and UG successions can be found in the main text. (d) Outcrop view of site MP‐II. (e) Close‐up of the sulfur‐bearing carbonates. Note clasts (cl) and cavernous fabric.

The purpose of this study is to better constrain the microbiome involved in the replacement of sulfate‐bearing evaporites under anoxic conditions. For this goal, lipid biomarker analysis combined with the measurement of compound‐specific δ^13^C values is applied after petrographic description and measurement of stable isotope compositions (δ^13^C, δ^18^O, and δ^34^S) of the mineral phases. Authigenic carbonate and native sulfur replacing gypsum were sampled in central Sicily from the previously unstudied Monte Palco ridge (Figure 1). At this site, the well‐exposed, in situ contact between gypsum and authigenic carbonate suggests that gypsum replacement is a young process. The geochemical results presented here agree with relatively recent secondary mineral formation, allowing us to describe the involved microbial communities in unprecedented detail.

## 2 Geological Background

The Monte Palco section is located in the Caltanissetta Basin in Sicily (Figure 1a). The Caltanissetta Basin is a thrust‐top basin that formed on the Gela Nappe, the outermost thrust sheet of the Apennine–Maghrebian thrust belt (Lickorish et al. 1999). The Gela Thrust Front is located at the southern margin of the basin (Figure 1a). The Caltanissetta Basin consists of a series of thrust‐controlled subbasins (Butler et al. 1995), filled with Miocene up to Middle Pleistocene deposits. The Miocene rock record of the Caltanissetta Basin encompasses the Messinian salinity crisis (MSC). During the MSC (5.97–5.33 Ma), tectonic and orbital forces caused major environmental, hydrological, and ecological changes in the Mediterranean Sea and resulted in the deposition of massive gypsum and halite in the Mediterranean deep and marginal basins (Roveri et al. 2014; Andreetto et al. 2021).

The succession exposed at the Monte Palco ridge records the acme and the last phase of the MSC (Manzi et al. 2009). The acme phase (5.60–5.55 Ma; Roveri et al. 2014) is recorded by brecciated carbonates belonging to the Calcare di Base (type 3 of Manzi et al. 2011), overlain by laminar cumulate gypsum deposits (Figure 1b,c), which are considered the lateral equivalent of halite deposits in the depocenters of the basins (Roveri et al. 2014). The brecciated carbonates and cumulate gypsum are overlain by the Upper Gypsum unit (Figure 1c; Manzi et al. 2009), representing the last phase of the MSC (5.55–5.33 Ma: Roveri et al. 2014). This unit displays a well‐defined lithological cyclicity, evidenced by the rhythmic alternation of marly layers and gypsum beds. The studied sulfur‐bearing carbonates are embedded in the uppermost part of the Upper Gypsum unit (Figure 1c); such carbonates are frequent in the Messinian sequence of Sicily and have commonly been categorized as Calcare di Base type 1 (Manzi et al. 2011). The Upper Gypsum unit is in turn overlain by the Pliocene foraminifera‐rich marine marly limestones of the Trubi Formation, which records the termination of the MSC (Figure 1c; Van Couvering et al. 2000).

## 3 Material and Methods

### 3.1 Preparation, Petrography, and Mineralogy

Field work was conducted at the Monte Palco ridge (37°25′15.7″ N, 13°59′00.2″ E) and a hill directly southwest of the main ridge (37°25′11.5″ N, 13°58′47.4″ E). A total of 12 carbonate rocks were sampled from two localities, referred to as “MP‐I” and “MP‐II” (Figure 1b; Table S1). For petrographic and geochemical reference, one marl sample from the Upper Gypsum cycle 4 (UG4) and one marly limestone sample from the Trubi Formation were collected from the vicinity of the two outcrops (Figure 1b). Thin sections were prepared from cut rock slices. All thin sections were partially stained with a combined potassium ferricyanide and alizarin red solution, dissolved in 0.1% HCl, to discern carbonate minerals (Füchtbauer 1988). Thin sections were studied under transmitted light on a Zeiss Axio Scope A1 microscope (Carl Zeiss AG, Oberkochen, Germany) mounted with a Canon EOS 1300D camera (Canon Inc., Tokio, Japan) at the Institut für Geologie, Universität Hamburg. Carbonate phase mineralogy of the sulfur‐bearing carbonates was confirmed with qualitative X‐ray diffraction (XRD) analysis, using a Panalytical X'Pert PRO diffractometer (CuKα radiation; Malvern Panalytical Ltd., Malvern, UK) at the Institut für Geologie, Universität Wien.

### 3.2 Stable Isotope Analyses

For the measurement of δ^13^C and δ^18^O values of carbonates, powders of different textural phases were collected by drilling samples with a handheld microdrill (Table S1). Isotope measurements were performed on a ThermoFisher Scientific 253plus gas isotope ratio mass spectrometer with a Kiel IV automated carbonate preparation device (Thermo Fisher Scientific Inc., Waltham, MA, USA) at the stable isotope laboratory of Zentrum für Marine Umweltwissenschaften (MARUM), Universität Bremen. The analytical error for the in‐house Solnhofen limestone standard (calibrated against the NBS 19 calcite standard) was 0.03‰ for δ^13^C and 0.06‰ for δ^18^O values. All carbon and oxygen stable isotope values are reported in per mil (‰) relative to the Vienna Pee Dee Belemnite (V‐PDB) standard.

For the analysis of δ^34^S values and carbonate‐associated sulfate (CAS) contents, subsamples were cut and manually ground down to fine whole rock powders. Easily soluble sulfate was removed from the powders by overnight soaking with a sodium chloride solution. The remaining carbonate was subsequently removed by dissolving it with hydrochloric acid. After dissolution, CAS was precipitated as barium sulfate by adding barium chloride to the acid‐insoluble residue (Gischler et al. 2020). CAS concentration was determined by weighing and δ^34^S measurements were performed on barium sulfate using an Elemental Analyzer (Elementar Pyro Cube; Elementar Analysensysteme GmbH, Langenselbold, Germany) coupled to an isotope ratio mass spectrometer (Elementar GeoVisION) at the University of Texas at El Paso, Texas, USA. Repeated analyses of in‐house and international standards showed a standard error (1*σ*) of less than 0.3‰ for δ^34^S values. All sulfur isotope values reported here are in ‰ relative to the Vienna‐Canyon Diablo Troilite (V‐CDT) standard.

For the measurement of δ^34^S values of native sulfur, *n*‐heptane and activated copper were repeatedly used on whole rock powders for the extraction of sulfur as Cu_x_S_x_. An acidic chromium reducible solution subsequently converted Cu_x_S_x_ to H_2_S (Fossing and Jørgensen 1989). H_2_S was precipitated as Ag_2_S powder by reaction with an AgNO_3_ solution (Geng et al. 2018). Following three times rinsing with MiliQ water and drying at 60°C, the Ag_2_S powder was reacted overnight at 310°C to gaseous SF_6_ using F_2_. Afterward, the SF_6_ was purified cryogenically and by gas chromatography. Analysis for δ^34^S on SF_6_ was performed with a ThermoFisher Scientific MAT‐253 dual inlet mass spectrometer at the Institut de Physique du Globe de Paris (Ono et al. 2006; Labidi et al. 2012). Analytical precision was ±0.10‰ based on repeated analysis of an in‐house SF_6_ standard versus the IAEA‐S‐1 international standard that underwent the same fluorination and purification procedures as the samples once turned into Ag_2_S (Brand et al. 2014). In addition, two volcanic native sulfur internal standards were chemically extracted, fluorinated, and purified following the same protocol as used for the samples, yielding δ^34^S = 7.9‰ and 8.0‰.

### 3.3 Extraction, Analysis, and Compound‐Specific Carbon Isotope Compositions of Lipid Biomarkers

Six samples of sulfur‐bearing carbonate (MP‐II‐1, MP‐II‐4, MP‐II‐11b, MP‐II‐12, MP‐II‐13b, and MP‐II‐14) were prepared, decalcified, and extracted for their lipid biomarker inventory, following the procedure described in Sabino et al. (2020). Rock surfaces were removed, the remaining carbonates were crushed, and subsequently cleaned by rinsing with a 10% HCl solution and acetone. After cleaning, the rock pieces were dissolved with a 10% HCl solution. The residual after dissolution was saponified with 6% KOH in methanol (MeOH), using ultrasonication at 80°C for 2 h. Lipids were subsequently extracted from the saponified sediment by repeated ultrasonication with dichloromethane (DCM):MeOH (3:1, v/v), until the solvent turned colorless. The total lipid extract (TLE) was subsequently separated, using a separatory funnel and MilliQ water. During separation, the transfer of fatty acids was ensured by keeping the pH constant at 2 by adding 10% HCl. An aliquot of the TLE was separated into a maltene and asphaltene fraction, using *n*‐hexane and DCM, respectively. Elemental sulfur was removed from the maltene fraction by reacting it with activated copper overnight. The maltene fraction was further split into individual fractions of increased polarity (hydrocarbons, ketones, alcohols, and fatty acids) using column chromatography (SPE Chromabond, NH2, 6 mL/500 mg). An aliquot of the alcohol fraction (50%) was derivatized for 2 h at 80°C by adding pyridine and N,O‐bis(trimethylsilyl)trifluoroacetamide (BSTFA; 1:1, v/v). The fatty acid fraction was derivatized for 1 h at 70°C by adding 10% boron trifluoride (BF_3_) in MeOH. After the derivatization of the acid fraction, *n*‐hexane and MilliQ water were added, and the organic supernatant containing the fatty acids was removed. Ether cleavage was performed on the underivatized aliquots of the alcohol fraction of samples MP‐II‐12, MP‐II‐13b, and MP‐II‐14. These samples were selected due to their high biomarker contents in the alcohol fractions. The resulting hydrocarbons were released by cleaving the side chains bound by ether bonds in archaeal and bacterial lipids such as glycerol dibiphytanyl glycerol tetraethers (GDGTs) and isoprenoidal and non‐isoprenoidal dialkyl glycerol diethers (DAGEs). Ether cleavage was achieved by treatment with hydrogen iodide and reduction with LiAlH_4_ (cf. Birgel et al. 2014). The desulfurization procedure of Schouten et al. (1993) was applied for the asphaltene fraction to release compounds from organic macromolecules that formed through intermolecular sulfurization (cf. Kutuzov et al. 2020). A detailed description was provided by Sabino et al. (2021). The resulting hydrocarbons after desulfurization were separated from the remaining polar compounds with a silica gel column. Lipid biomarkers were identified using a Thermo Scientific Trace GC Ultra gas chromatograph (GC) coupled to a Thermo Scientific DSQ II mass spectrometer. Biomarker quantification was done using a Thermo Scientific Trace GC 1310 with a flame ionization detector. The internal standards added for quantification were 5α(H)‐cholestane for hydrocarbons, 1‐nonadecanol and *n*‐C_18_/*n*‐C_18_‐dialkyl glycerol diether for the alcohols, 2me‐C_18_ fatty acid for the carboxylic acids, and 10me‐C_19_ alkane for the asphaltenes. The GC temperature program for all measurements was set to hold for 3 min at 50°C, subsequently ramping up to 230°C at 5°C/min, and at last to 325°C at 6°C/min, which was held for 25 min.

Compound‐specific δ^13^C values for the hydrocarbons, alcohols, fatty acids, and ether cleaved products were obtained with an Agilent 6890 GC (Agilent Technologies Inc., Santa Clara, CA, USA) coupled to a Thermo Finnigan Combustion III interface and a Thermo Finnigan Delta Plus isotope ratio mass spectrometer at the Institut für Geologie, Universität Hamburg. The same temperature program as described above was used. For the hydrocarbons derived after desulfurization of the asphaltenes, δ^13^C measurements were conducted with an Agilent 6890 GC coupled via a Thermo Finnigan Combustion III to a Thermo Finnigan MAT 253 isotope ratio mass spectrometer at MARUM, Universität Bremen. For these δ^13^C measurements, the GC temperature program was set to hold for 2 min at 50°C, subsequently ramping up to 320°C at 5°C/min, at which it was held for 35 min. All compound‐specific δ^13^C values are reported in ‰ relative to the V‐PDB standard and were corrected for derivatization when applicable. The average deviation of δ^13^C values for repeated measurements was less than 1‰.

GDGTs were measured with normal‐phase HPLC using aliquots of the maltene fractions, which were dissolved in *n*‐hexane:propan‐2‐ol (99:1, v/v). Measurements were performed on a Waters Alliance 2690 HPLC system (Waters Cooperation, Milford, MA, USA) fitted with a Grace Prevail Cyano column (150 · 2.1 mm i.d., 3 μm particle size; Thermo Fisher Scientific, Waltham, MA, USA) and a security guard column cartridge of the same material at the Institut für Geowissenschaften, Universität Kiel. For settings used to separate target compounds, see Baumann et al. (2018). C_46_‐GDGT was used as an internal standard for quantification of isoprenoidal GDGTs.

## 4 Results

### 4.1 Field Observations

The Monte Palco section is characterized from the base to the top by a succession of thick beds of brecciated carbonates (roughly 30 m in total), 5 m of laminar cumulate gypsum (commonly referred to as balatino), a roughly 45 m thick cyclic succession of gypsum beds and thin marly layers, and at least 10 m of foraminifera‐rich marly limestones (Figure 1b,c). The units are assigned to the Calcare di Base type 3, Upper Gypsum unit (UG), and Trubi Formation by Manzi et al. (2009), respectively. Six gypsum cycles are observed in the UG (Figure 1b,c). The first cycle (UG1) consists of laminar cumulate gypsum and banded selenite. The following cycles all have a roughly 1–2 m thick layer of marlstone at the base, overlain by a bed of bottom‐grown gypsum. The gypsum bed of UG2 consists of massive selenite, whereas the beds of UG3–UG6 are made up of banded selenite with characteristic domed structures. Every UG bed is roughly 10 m thick. The thickness of UG6, however, could not be determined because it is truncated by a NE–SW striking fault that juxtaposes the Trubi Formation with the different UG cycles (Figure 1b). The studied sulfur‐bearing carbonates form an irregular‐shaped body, about 7 m high and 5 m wide, aligned along the fault, at the contact between the UG and the Trubi Formation (Figure 1d). These rocks show a brecciated texture and a cavernous fabric (Figure 1e). The elongated prismatic habit of most of the empty cavities suggests the former presence of gypsum crystals.

### 4.2 Petrography and Mineralogy

**FIGURE 2:**
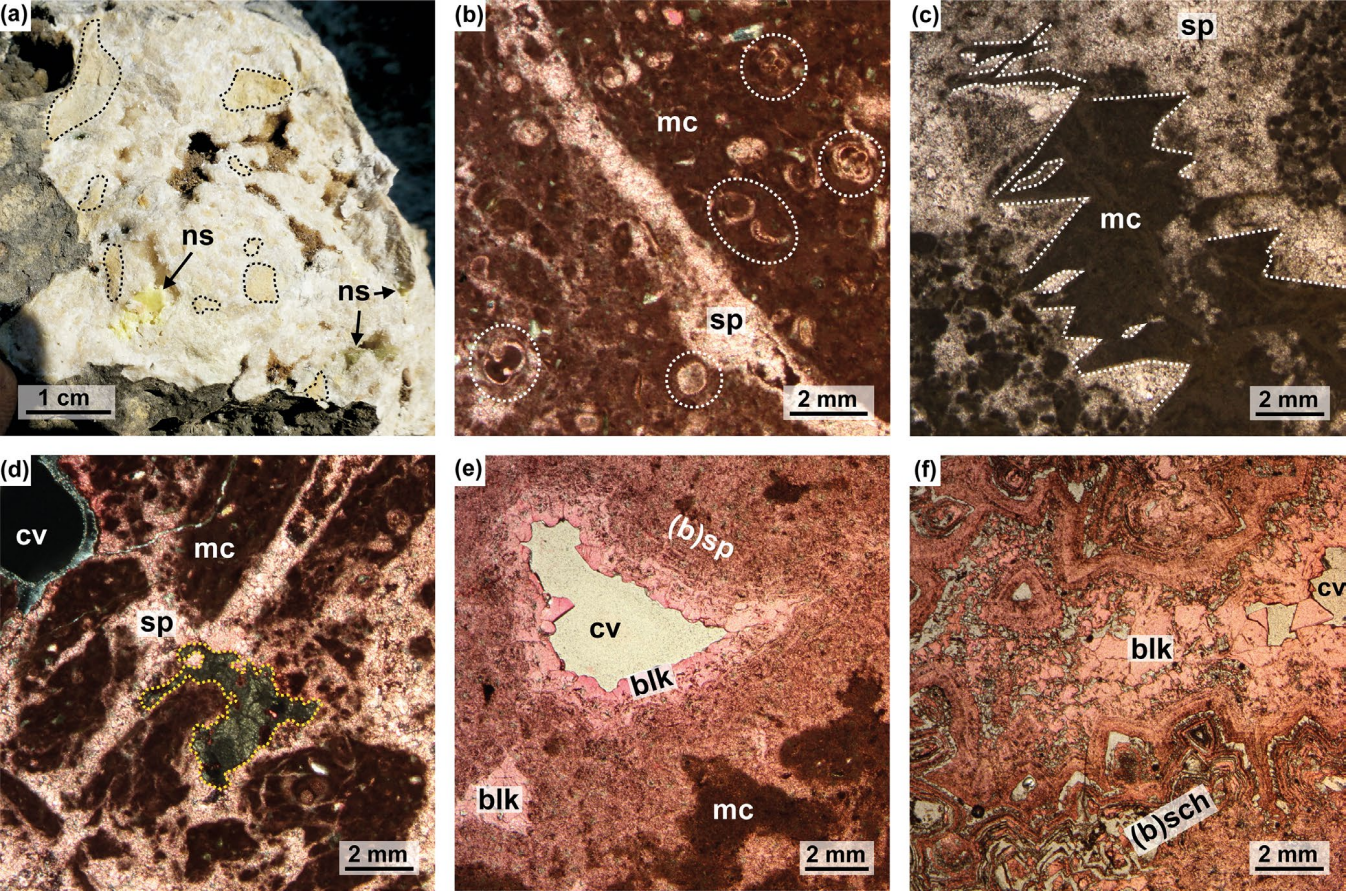
Petrography of the sulfur‐bearing carbonates from the sampling site “MP‐II”. (a) A hand sample of sulfur‐bearing carbonate. Clasts are encircled by black dotted lines. (b) Sparite vein crosscutting a micrite clast with foraminifera microfossils (encircled with white dashed line); sample MP‐II‐13b, cross‐polarized light. (c) Pseudomorphs after gypsum (white dashed line) filled with carbonate; sample MP‐II‐11b, plane‐polarized light. (d) Micrite clasts, (micro)sparite cement, and elemental sulfur (yellow dashed line); sample MP‐II‐13b, cross‐polarized light. (e) Blocky calcite and (micro)sparite cement with banded microtexture surrounding a cavity; sample MP‐II‐14, plane‐polarized light. (f) Blocky calcite and banded scalenohedral calcite; sample MP‐II‐1, plane‐polarized light. Thin sections (b), (d), (e), and (f) stained with alizarin red‐S, resulting in pink stain of calcite. (b) = banded; blk = blocky calcite; cv = cavity; mc = micrite; ns = native sulfur; sch = scalenohedral calcite; sp = (micro)sparite.

The sulfur‐bearing carbonates consist of matrix‐supported breccias containing millimeter‐ to centimeter‐sized clasts of mudstone and wackestone (Figure 2a), composed of aphanitic micrite, clotted micrite, and peloidal micrite (Figure 2a). Clasts do not contain sulfur. Some clasts, consisting of foraminifera‐rich wackestones, were probably sourced from the Trubi Formation (e.g., sample MP‐II‐13b; Figure 2b). Other clasts are typified by silt‐sized quartz grains and pseudomorphs after gypsum, ranging in size from 500 μm up to several mm (e.g., sample MP‐II‐11b; Figure 2c). Pseudomorphs are roughly lenticular to lozenge‐shaped (Figure 2c).

The matrix of the breccia is entirely made up of calcitic micrite and (micro)sparite that lacks any internal lamination (Figure 2d,e). The same types of calcite cements fill the gypsum pseudomorphs within the clasts (Figure 2c). Cavities are common in the sulfur‐bearing carbonates; they are partially filled with isopachous banded and zoned scalenohedral calcite and blocky calcite (Figure 2e,f). Native sulfur crystals are either randomly dispersed in the calcite cement of the matrix or fill residual porosity (Figure 2d). Occasionally, celestine has also been found filling pore space.

### 4.3 Stable Isotope Compositions

**FIGURE 3:**
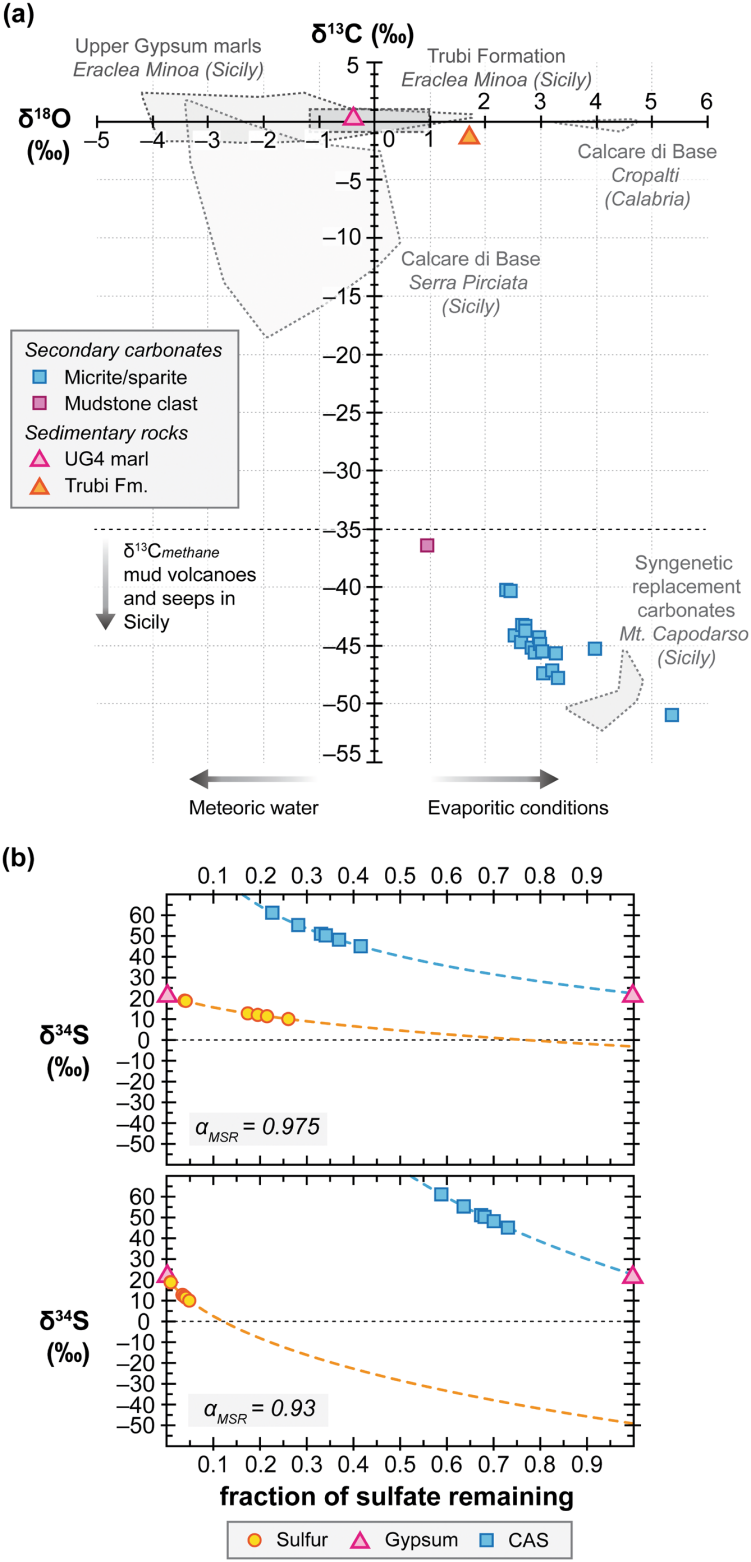
Stable isotope composition for bulk rocks and selected carbonate phases from the study area. (a) Carbon and oxygen stable isotopes of carbonates. Gray dotted fields denote literature data for different stratigraphic units from Sicily and Calabria (both southern Italy). Sections specified in italics are after Birgel et al. (2014) and Caruso et al. (2015) for Calcare di Base, after Longinelli (1979) for the UG, after Pierre et al. (2006) for Trubi Formation and after Ziegenbalg et al. (2010) for syngenetic sulfur‐bearing carbonates (Monte Capodarso, Sicily). Black horizontal dotted line denotes least ^13^C‐depleted value of methane from seeps and mud volcanoes in Sicily, based on values from Grassa et al. (2004) and Tassi et al. (2012). The arrows at the bottom reflect the common oxygen isotope trend in carbonates for relevant hydrological conditions. All other samples are from this study (as indicated in the legend). (b) Rayleigh plots of sulfur isotope evolution for δ^34^S values of native sulfur and CAS (*y*‐axis), as a function of sulfate fraction remaining (*x*‐axis), assuming UG gypsum as sulfate source (δ^34^S_average_ = 22.4‰; García‐Veigas et al. 2018). The upper plot uses 25‰ as fractionation of microbial sulfate reduction for the fractionation factor (*α*_MSR_ = 0.975) and the lower plot uses 70‰ (*α*_MSR_ = 0.93). Calculated sulfur isotope composition curves for native sulfur and CAS are shown as yellow and blue dashed lines, respectively.

The micrite and (micro)sparite cements of the sulfur‐bearing carbonates show negative δ^13^C values ranging from −51.0‰ to −40.2‰ and positive δ^18^O values ranging from 2.4‰ to 5.4‰ (Figure 3a; Table S1). A mudstone clast in sample MP‐II‐11b yielded a δ^13^C value of −36.4‰ and a δ^18^O value of 0.9‰. The marls of the UG4 cycle and of the Trubi Formation show δ^13^C values of 0.2‰ and −1.4‰ and δ^18^O values of −0.4‰ and 1.7‰, respectively. Contents of CAS in the sulfur‐bearing carbonates range from 1185 ppm to 2753 ppm (Table S1). The sulfur‐bearing carbonates have high δ^34^S_CAS_ values ranging from 45.1‰ to 61.1‰ and δ^34^S_native sulfur_ values ranging from 10.1‰ to 18.8‰ (Figure 3b).

Fractions of sulfate remaining were subsequently calculated with an adapted Rayleigh equation for fractional distillation in a closed system (Figure 3b; Mariotti et al. 1981; Hayes 1983; Gomes and Hurtgen 2015), using two different fractionation factors (based on the minimum offset found between δ^34^S values of CAS and S_0_ (Table S1) and the maximum offset for microbial sulfate reduction; Brunner and Bernasconi 2005; Canfield et al. 2010; Sim et al. 2011) and the average δ^34^S value of sulfate in UG (22.4‰; García‐Veigas et al. 2018) in the following equations:

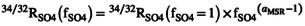




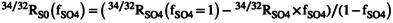

In the equations, f_SO4_ corresponds to the fraction of sulfate remaining in the system, where f_SO4_ = 1 corresponds to a full sulfate reservoir (no sulfate has been removed by microbial sulfate reduction) and f_SO4_ = 0 corresponds to no sulfate remaining (all sulfate has been removed by microbial sulfate reduction). Functions ^34/32^R_SO4_(f_SO4_) and ^34/32^R_S0_(f_SO4_) describe the evolution of the isotopic ratio of respectively sulfate during its consumption by microbial sulfate reduction and native sulfur produced by microbial sulfate reduction (either by direct reduction of sulfate to native sulfur or by oxidation of reduced sulfur species without significant fractionation during oxidation). *α*_MSR_ corresponds to the fractionation factor of microbial sulfate reduction, for which fractionations of 25‰ (*α*_MSR_ = 0.975; upper plot in Figure 3b) and 70‰ (*α*_MSR_ = 0.93, lower plot in Figure 3b) were used. Using the minimum fractionation and the δ^34^S values of sulfur species obtained from the carbonates, the δ^34^S values reflect that 4%–26% of sulfate remained during the precipitation of respective native sulfur and 23%–42% when respective sulfate was captured within the carbonates (CAS). Using the maximum fractionation, 1%–5% of sulfate remained for native sulfur and 59%–73% for CAS. Alternatively, it has been proposed that a Rayleigh distillation model may not be entirely appropriate because in proximity to gypsum deposits, there is a resupply of sulfate, and any fluid flow would be expected to entrain produced sulfide and residual sulfate, making a closed system argument difficult to uphold (Labrado et al. 2019). Instead, an isotope mass balance approach would be more appropriate:

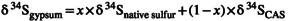

In this equation, *x* corresponds to the degree of openness, with *x* = 0 meaning open (sulfate escapes and δ^34^S_CAS_ = δ^34^S_gypsum_) and *x* = 1 meaning closed (sulfate converts to native sulfur and δ^34^S_native sulfur_ = δ^34^S_gypsum_). This results in a sulfur isotope fractionation of ca. 38‰, with 69%–95% of sulfur released by gypsum dissolution ending up as native sulfur. Thus, either approach suggests a high degree of conversion of sulfate to native sulfur and insignificant sulfate removal.

### 4.4 Lipid Biomarker Inventory

#### 4.4.1 Desulfurized Asphaltenes

**FIGURE 4:**
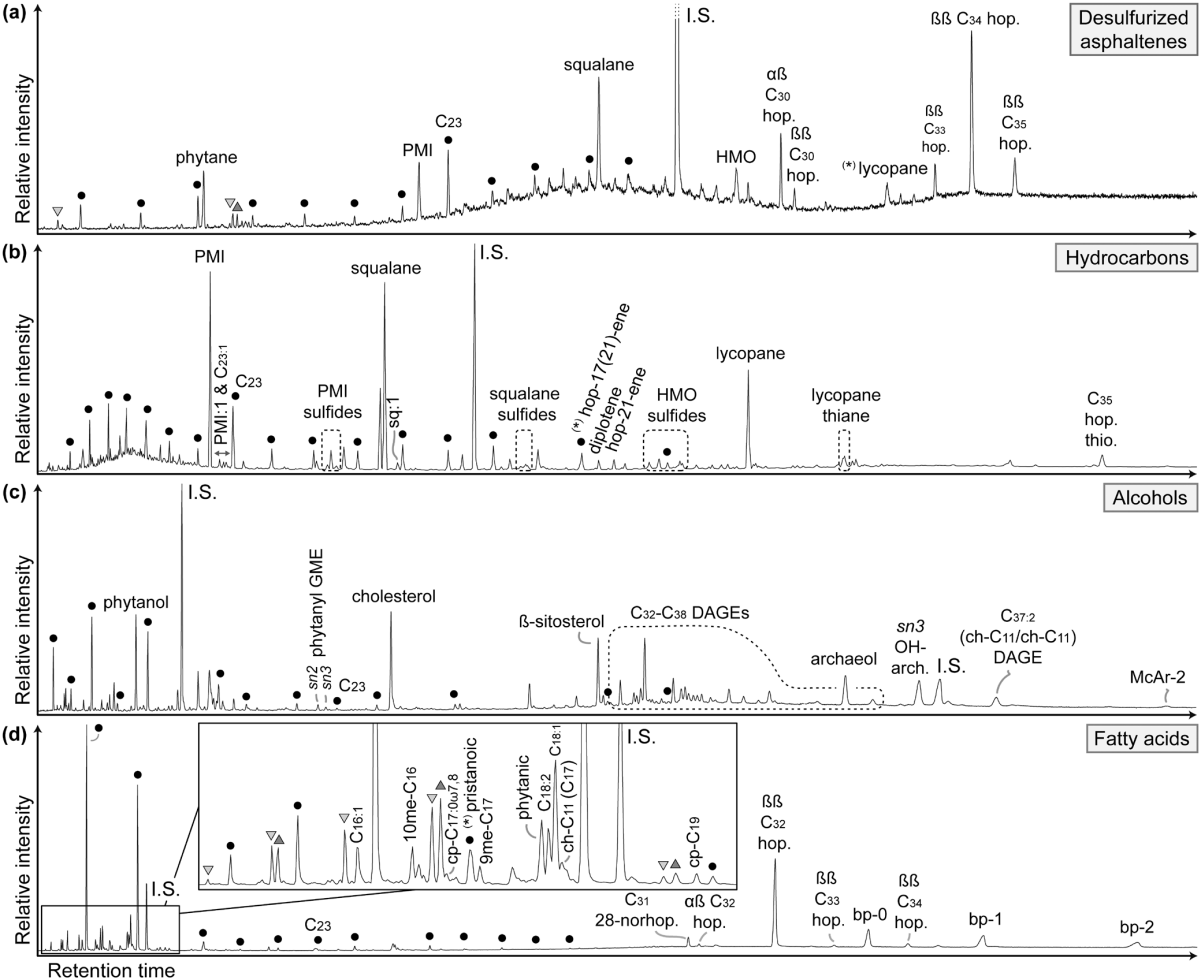
Partial gas chromatograms (total ion current) from sample MP‐II‐13b; (a) desulfurized asphaltene fraction, (b) hydrocarbon fraction, (c) alcohol fraction, and (d) fatty acid fraction. Black circles denote *n*‐alkyl compounds in the respective fractions, triangle symbols downward (light gray) and upward (dark gray) denote *iso*‐compounds and *anteiso*‐compounds, respectively. Star in parentheses denotes co‐elution of compounds. The chromatograms of (a) and (b) are misaligned in respect to each other, due to different zoom for better graphical representation of (a). Abbreviations used that are not specified in the text: Hop. = hopane (desulfurized fraction and hydrocarbon fraction) and homohopanoic acid (fatty acid fraction), respectively, αβ and ββ = 17α(H),21β(H)‐ and 17β(H),21β(H)‐, respectively (both desulfurized and fatty acid fraction); thio. = thiophene (hydrocarbon fraction); *sn3* OH‐arch. = *sn3*‐hydroxyarchaeol (alcohol fraction); ch‐ = cyclohexyl (both alcohol and fatty acid fraction); cp‐ = cyclopropyl‐, C_31_ 28‐norhop. = C_31_ 28‐norhopanoic acid; bp‐0, ‐1 and ‐2 = acyclic, monocyclic and bicyclic biphytanic diacid (fatty acid fraction); DAGE = dialkyl glycerol diether; GME = glycerol monoalkyl ether; HMO = 2,6,10,14,19,23,27‐heptamethyloctacosane; I.S. = internal standard; McAr = macrocyclic archaeol; me = methyl; PMI = 2,6,10,15,19‐pentamethylicosane.

**FIGURE 5:**
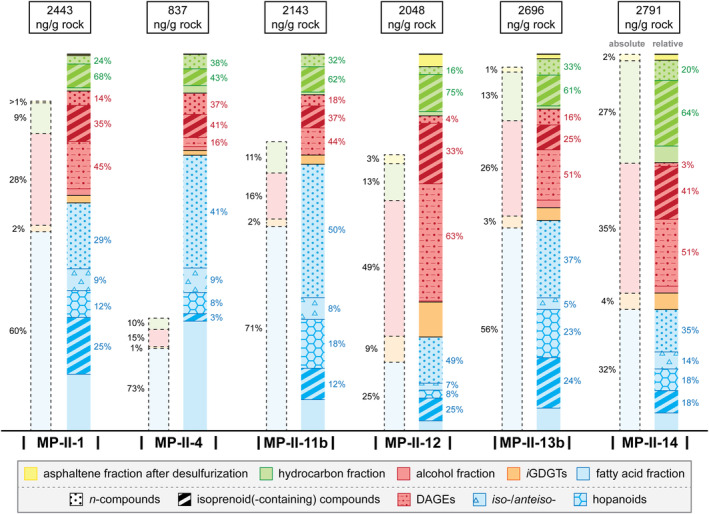
Total contents (ng/g rock) and relative percentages of the respective lipid fractions from the sulfur‐bearing carbonates. The left columns denote the percentages of the respective fractions, displayed in black letters. Absolute column heights are relative to the total lipid biomarker content of sample MP‐II‐14, which has the highest lipid content. The right columns are the percentages of specific compound groups in the various fractions. Colors and symbols of the fractions are described in the legend. Compound groups and abbreviations are specified in the text.

The desulfurization of asphaltenes produced low amounts of hydrocarbons (from 11 ng/g rock in sample MP‐II‐1 to 69 ng/g rock in sample MP‐II‐12) that include *n*‐alkanes, acyclic isoprenoids, and hopanes (Figures 4a and 5; Table S2). The lipid contents of this fraction correspond to 1–3 wt% of the total lipid inventory in samples MP‐II‐12, MP‐II‐13b, and MP‐II‐14. Saturated acyclic isoprenoids are the most abundant compounds after desulfurization, ranging from 32% of all desulfurized asphaltenes in MP‐II‐12 to 50% in MP‐II‐14 (not shown in Figure 5). Among the isoprenoids, the tail‐to‐tail linked C_30_ isoprenoid 2,6,10,15,19,23‐hexamethyltetracosane is most abundant (squalane; 37% of all isoprenoids in MP‐II‐12 and MP‐II‐14 and up to 44% in MP‐II‐1). Other isoprenoids are the head‐to‐tail linked C_20_ isoprenoid 2,6,10,14‐tetramethylhexadecane (phytane; 12% in MP‐II‐12 to 17% in MP‐II‐13b), the tail‐to‐tail linked C_25_ isoprenoid 2,6,10,15,19‐pentamethylicosane (PMI; 18% in MP‐II‐1 to 25% in MP‐II‐13b), the tail‐to‐tail linked C_35_ isoprenoid 2,6,10,14,19,23,27‐heptamethyloctacosane (HMO; 14% in MP‐II‐14 to 18% in MP‐II‐1 and MP‐II‐13b), and the tail‐to‐tail linked C_40_ isoprenoid 2,6,10,14,19,23,27,31‐octamethyldotriacontane (lycopane; traces in MP‐II‐1 to 15% in MP‐II‐12). Lycopane partially co‐elutes with the *n*‐C_35_ alkane. Saturated *n*‐alkanes are the second most abundant group of compounds (20% of all compounds in MP‐II‐14 to 42% in MP‐II‐1). Among them, the *n*‐C_23_ alkane is most abundant, ranging from 20% in MP‐II‐13b to 69% in MP‐II‐14. Hopanes with 30–35 carbons are also present (19% of all compounds in MP‐II‐1 to 33% in MP‐II‐12). The most abundant hopanes are 17(H)β,21(H)β‐C_34_ hopane (50% of hopanes after desulfurization in MP‐II‐14 to 68% in MP‐II‐1) and 17(H)α,21(H)β‐C_30_ hopane (17% in MP‐II‐13b to 32% in MP‐II‐1).

#### 4.4.2 Free Hydrocarbons

The free hydrocarbons include saturated isoprenoids (both non‐sulfurized and sulfurized) and saturated *n*‐alkanes (Figure 4b), with a total content ranging from 87 ng/g rock in sample MP‐II‐4 to 760 ng/g rock in sample MP‐II‐14 (Figure 5; Table S2). On average, hydrocarbons constitute 11% of the total lipid inventory, with the notable exception of sample MP‐II‐14, which has 27% of the total inventory (Figure 5). Most saturated hydrocarbon isoprenoids are non‐sulfurized acyclic isoprenoids, ranging from 65% of all saturated hydrocarbon isoprenoids in sample MP‐II‐14 to 88% in sample MP‐II‐4. Of the non‐sulfurized acyclic isoprenoids, squalane (33% of these isoprenoids in MP‐II‐1 to 58% in MP‐II‐11b) and PMI (22% in MP‐II‐11b to 50% in MP‐II‐1) are the most prominent compounds in all hydrocarbon fractions (Figure 4b), followed by lycopane (15% in MP‐II‐1 to 26% in MP‐II‐12). Other, minor head‐to‐tail linked isoprenoids are 2,6,10,14‐tetramethylpentadecane (pristane) in samples MP‐II‐1 and MP‐II‐4 and phytane in all samples except MP‐II‐11b. Aside from saturated non‐sulfurized isoprenoids, minor amounts of monounsaturated squalenes were found in all samples, accompanied by minor amounts of monounsaturated PMIs.

Other saturated hydrocarbon isoprenoids are organic sulfur compounds (OSCs), which were identified by their molecular masses, relative retention times (Figure 4b), and by comparison with mass spectra of other squalene‐derived and lycopane‐derived sulfides (Figure S1; cf. Grice et al. 1998; Wu et al. 2020). The inventory of OSC isoprenoids among the hydrocarbons is similar to the inventory of the asphaltenes after desulfurization. Among the OSCs are exclusively tail‐to‐tail linked isoprenoids including PMI, squalane, and HMOs as thianes and thiolanes (Figure S1a–c, respectively), whereas lycopane is only found as thiane (Figure S1d). Other, non‐isoprenoid OSCs include C_32_ and C_35_ thiophene hopanes (cf. Valisolalao et al. 1984). Among the free *n*‐alkanes, *n*‐C_23_ alkane is most abundant in all samples (15% of *n*‐alkanes in MP‐II‐13b to 52% in MP‐II‐12), except for sample MP‐II‐4 where *n*‐C_31_ alkane is most abundant (22%). All samples also contain a minor amount of monounsaturated *n*‐C_23_ alkane. In all samples, minor amounts of hopanoids are found (1%–2% of free hydrocarbons in all samples), except for traces in MP‐II‐4. The hopanoids consist of unsaturated C_30_ hopanes, specifically hop‐17(21)‐ene (partial co‐eluting with *n*‐C_31_ alkane in all samples), hop‐22(29)‐ene (diploptene), and hop‐21‐ene.

#### 4.4.3 Alcohols and GDGTs

The alcohol fractions contain saturated *n*‐alcohols, a series of non‐isoprenoid dialkyl glycerol diether lipids (DAGEs; Figure 4c), isoprenoid alcohols including abundant diphytanyl glycerol diether lipids, and glycerol dibiphytanyl glycerol tetraethers (GDGTs; Table S2). The total content of the alcohol fraction (i.e., including GDGTs) accounts for 138 ng/g rock in MP‐II‐4 to 1201 ng/g rock in MP‐II‐12, which represent 17%–59% of the total lipid inventory, respectively (Figure 5; Table S2).

Major isoprenoid compounds in the alcohol fraction are two diether lipids: the diphytanyl (C_20_‐C_20_) glycerol diether archaeol and *sn3*‐hydroxyarchaeol, together amounting to 73% of the isoprenoid alcohols (excluding isoprenoid GDGTs) in MP‐II‐13b to 96% in MP‐II‐12. Another, minor diether isoprenoid is macrocyclic archaeol (McAr) with two cyclopentane rings (McAr‐2; cf. Stadnitskaia et al. 2003), which is especially abundant in sample MP‐II‐13b (4% of the isoprenoid alcohols). Other minor isoprenoids are two phytanyl glycerol monoethers, *sn2*‐ and *sn3*‐phytanyl glycerol monoether. Additional compounds present are isoprenoid GDGTs (*i*GDGTs) with two biphytane chains with variable cyclization (GDGT‐0 to −4; cf. Schouten et al. 2013). Their content ranges from 11 ng/g rock in MP‐II‐4 to 192 ng/g rock in MP‐II‐12, or 1%–9% of the total biomarker content, respectively (Figure 5). GDGT‐0 (19% of GDGTs in MP‐II‐12 to 31% in MP‐II‐11b) and GDGT‐2 (22% in MP‐II‐4 to 36% in MP‐II‐12) are the most abundant *i*GDGTs, followed by GDGT‐1 (16% in MP‐II‐13b and MP‐II‐14 to 21% in MP‐II‐4), GDGT‐3 (16% in MP‐II‐11b and MP‐II‐12 to 20% in MP‐II‐1), and minor GDGT‐4 (7% in MP‐II‐12 to 11% in MP‐II‐13b).

Similarly abundant as the isoprenoid diether lipids, a wide variety of DAGEs is present in all samples (Figure 4c; Table S2). Overall, DAGEs in the sulfur‐bearing carbonates have 32–38 carbons (including the three carbons from the glycerol moieties). DAGEs with 33 carbons are most abundant (32% of DAGEs in MP‐II‐13b to 58% in MP‐II‐1), followed by those with 34 carbons (14% in MP‐II‐13b to 40% in MP‐II‐4) and either 32 carbons (11% in MP‐II‐11b to 14% in MP‐II‐12 and MP‐II‐14) or 35 carbons (11% in MP‐II‐11b to 23% in MP‐II‐13b). The most abundant individual DAGE is C_33_ (*anteiso*‐C_15_/*anteiso*‐C_15_) DAGE (19% of DAGEs in MP‐II‐13b to 42% in MP‐II‐1). The alcohol fractions of samples MP‐II‐12, MP‐II‐13b, and MP‐II‐14 were ether cleaved to identify the alkyl chains included in the DAGEs. Aside from *n*‐alkyl chains, methylated (including cyclopropyl‐derived, see below) alkyl chains are also common (38% of DAGE‐derived products in MP‐II‐13b to 73% in MP‐II‐12; Table S2). Terminally branched alkanes derived from DAGEs are particularly abundant in MP‐II‐14 and MP‐II‐12 (57% and 63% of DAGE‐derived products, respectively), but less abundant in MP‐II‐13b (15%). *Anteiso*‐C_15_ alkane is the most abundant terminally branched alkane derived from DAGEs in MP‐II‐14 and MP‐II‐12 (72% and 73%, respectively). Other methyl compounds present are abundant 10me‐C_16_ alkane in all samples and minor dimethyl‐C_15_ and 6me‐C_14_ alkanes in MP‐II‐12 and MP‐II‐14. Other compounds produced by ether cleavage of DAGEs are cyclic alkanes (Table S2), including cyclohexyl‐C_11_ (C_17_) alkane (7% of DAGE‐derived products in MP‐II‐12 to 20% in MP‐II‐13b). DAGEs with cyclopropyl chains are likely present in samples MP‐II‐12 and MP‐II‐14, as evidenced by minor 4me‐ and 5me‐C_15_ alkanes in all samples (co‐eluting in relatively similar abundance, likely derived from cyclopropyl‐C_16_), and 8me‐ and 7me‐C_18_ alkanes in all samples (co‐eluting in relatively similar abundance, likely derived from cyclopropyl‐C_19_). Based on the combined results of mass spectra, ^13^C‐depletions, and abundances of both DAGEs and ether‐cleaved products, as well as their relative elution times and structural elution behaviors (Kovats Indices; cf. Pancost, Bouloubassi, et al. 2001; Bradley et al. 2009; Vinçon‐Laugier et al. 2016), novel DAGEs were tentatively identified (Figure S2).

#### 4.4.4 Fatty Acids

The fatty acid fractions contain saturated *n*‐fatty acids, terminally branched *iso*‐ and *anteiso*‐fatty acids, hopanoic acids, and isoprenoid acids (Figure 4d) and have the highest total biomarker contents, ranging from 508 ng/g rock in sample MP‐II‐12 to 1515 ng/g rock in sample MP‐II‐11b (Figure 5; Table S2). In most samples, fatty acids represent more than 50% of the total lipid inventory; only in samples MP‐II‐12 and MP‐II‐14 fatty acids comprise 25% and 32% of all lipids, respectively. The *n*‐C_16_ and *n*‐C_18_ fatty acids are the most abundant compounds among the saturated *n*‐fatty acids in all samples (together 53% of saturated *n*‐fatty acids in MP‐II‐4 to 92% in MP‐II‐12; Figure 4d). Terminally branched fatty acids range from *iso*‐C_13_ to *anteiso*‐C_19_. Among them, the most abundant are fatty acids with 15 and 17 carbons (both *iso*‐ and *anteiso‐*; accounting together for 58% of all terminally branched fatty acids in MP‐II‐4 to 76% in MP‐II‐12). Other fatty acids identified include minor amounts of various cyclic (cyclopropyl and cyclohexyl) fatty acids and hydroxy‐fatty acids. Cyclopropyl‐C_17:0ω7,8_ and cyclopropyl‐C_19:0_ fatty acids are present in all samples. Minor cyclohexyl‐C_11_ (C_17_) fatty acid (partially) co‐elutes with C_18:1_ fatty acid in samples MP‐II‐13b and MP‐II‐14.

Hopanoic acids range from 29 to 34 carbons. Most abundant are homohopanoic acids with 31–34 total carbons (70% of hopanoic acids in MP‐II‐12 to 100% in MP‐II‐1 and MP‐II‐4). The majority of homohopanoic acids are 17β(H),21β(H)‐isomers (90% in MP‐II‐12 to 100% in MP‐II‐4). Among the hopanoic acids, 17β(H),21β(H)‐C_32_ homohopanoic acid is most abundant (35% of hopanoic acids in MP‐II‐12 to 100% in MP‐II‐4).

Isoprenoid acids almost exclusively consist of phytanic acid and biphytanic diacids (98% of isoprenoid acids in MP‐II‐13b to 100% in MP‐II‐4 and MP‐II‐14). Biphytanic diacids include acyclic, monocyclic (biphytane chain with one cyclopropane moiety), and bicyclic (biphytane chain with two cyclopropane moieties) biphytanic diacids. Of these three, the bicyclic biphytanic diacid is slightly less abundant, whereas acyclic and monocyclic biphytanic diacids are relatively similar in abundance in samples MP‐II‐4, MP‐II‐11b, MP‐II‐13b, and MP‐II‐14. Minor amounts of pristanoic acid, partially co‐eluting with the *n*‐C_17_ fatty acid in samples MP‐II‐1, MP‐II‐11b, MP‐II‐12, and MP‐II‐13b, were also found.

### 4.5 Compound‐Specific δ^13^C Values of Lipid Biomarkers

**FIGURE 6:**
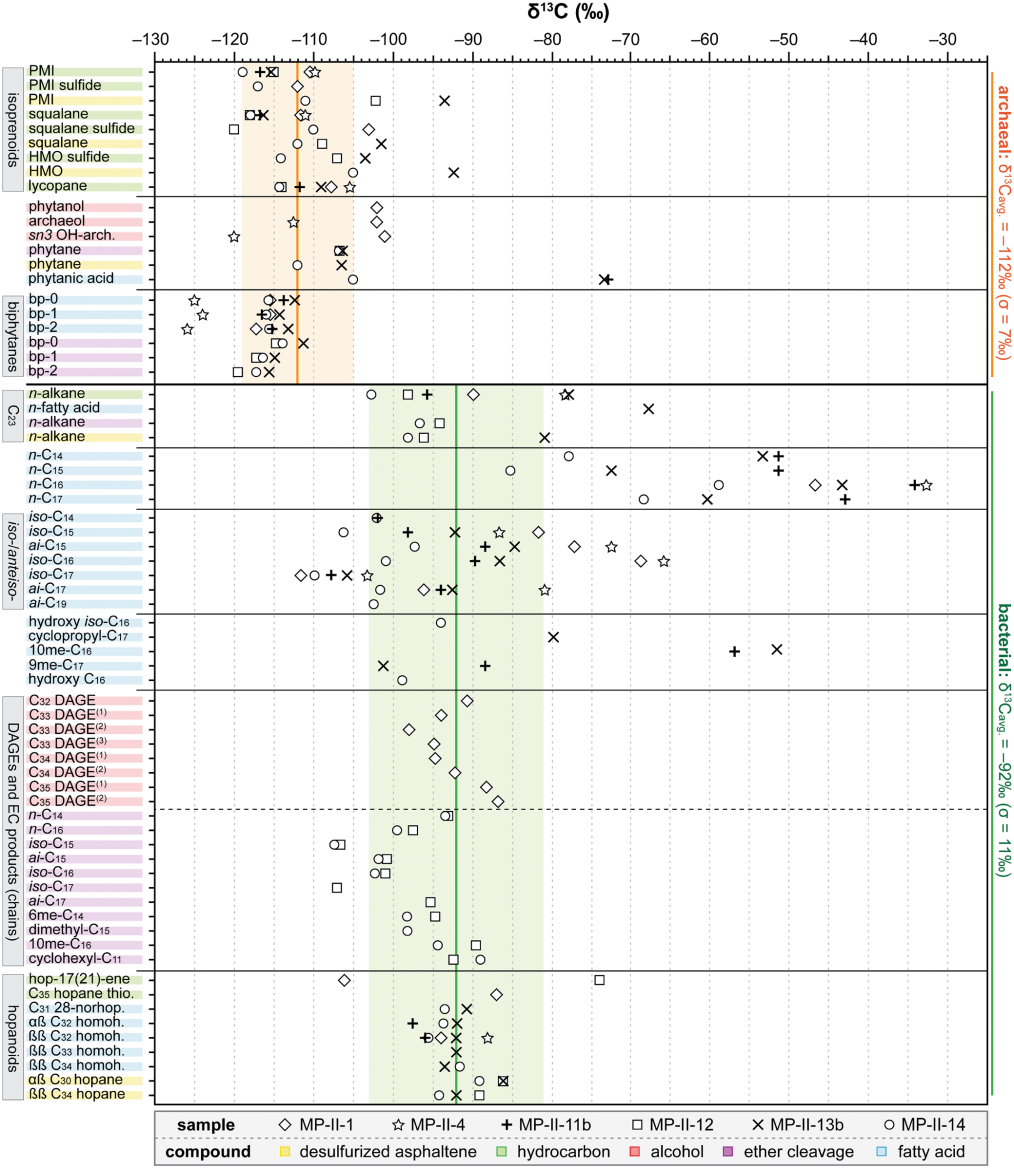
Compound‐specific carbon isotope composition for selected biomarkers in the sulfur‐bearing carbonates. All δ^13^C values are in ‰ versus V‐PDB. The thick horizontal black line separates archaeal‐derived lipids (upper part) and bacterial‐derived lipids (lower part). The orange (archaea) and green vertical lines (bacteria) display average δ^13^C values for the respective lipids. The orange and green horizontal fields display the standard deviation (1*σ*) of the average δ^13^C values. Gray bars on the left axis denote general compound groups and the colored bars denote the specific fraction for displayed compound. Abbreviations used that are not previously specified are PMI = 2,6,10,15,19‐pentamethylicosane, HMO = 2,6,10,14,19,23,27‐heptamethyloctacosane, *ai‐* = *anteiso*‐branched, cyclopropyl‐C_17_ = cyclopropyl‐C_17:0ω7,8_ fatty acid, C_32_ DAGE = C_32_ (*n*‐C_14_/*anteiso*‐C_15_) DAGE, C_33_ DAGE^(1)^ = C_33_ (6me‐C_14_/*iso*‐C_15_) DAGE co‐eluting with C_33_ (*iso*‐C_15_/*iso*‐C_15_) DAGE, C_33_ DAGE^(2)^ = C_33_ (*iso*‐C_15_/*anteiso*‐C_15_) DAGE, C_33_ DAGE^(3)^ = C_33_ (*anteiso*‐C_15_/*anteiso*‐C_15_) DAGE, C_34_ DAGE^(1)^ = C_34_ (*iso*‐C_16_/*anteiso*‐C_15_) DAGE co‐eluting with C_34_ (dimethyl‐C_15_/*n*‐C_14_) DAGE, C_34_ DAGE^(2)^ = C_34_ (*n*‐C_16_/*anteiso*‐C_15_) DAGE, C_35_ DAGE^(1)^ = C_35_ (10me‐C_16_/*anteiso*‐C_15_) DAGE and C_35_ DAGE^(2)^ = C_35_ (*anteiso*‐C_17_/*anteiso*‐C_15_) DAGE, and homoh. = homohopanoic acid.

Isoprenoids are the most strongly ^13^C‐depleted lipids in the sulfur‐bearing carbonates of Monte Palco, with an average δ^13^C value of −112‰ (*σ* = 7‰; “archaeal” in Figure 6). The range of δ^13^C values extends from −126‰ for bicyclic biphytanic diacid (MP‐II‐4) to −92‰ for HMO after desulfurization (MP‐II‐12). Another group of ^13^C‐depleted lipids is the DAGEs, with δ^13^C values from −98‰ to −87‰ (measured in MP‐II‐1). DAGE‐derived products after ether cleavage in other samples show similar ^13^C‐depletions, ranging from −107‰ for *iso*‐C_15_ (MP‐II‐12 and ‐14) and for *iso*‐C_17_ (MP‐II‐12) to −89‰ for cyclohexyl‐C_11_ (MP‐II‐14). Hopanoid compounds have ^13^C‐depletions similar to the DAGEs, with δ^13^C values from −97‰ for 17α(H),21β(H)‐C_32_ homohopanoic acid (MP‐II‐11b) to −88‰ for 17β(H),21β(H)‐C_32_ homohopanoic acid (MP‐II‐4). The δ^13^C values of hop‐17(21)‐ene fall slightly outside this range, with δ^13^C values of −106‰ (MP‐II‐1) and −74‰ (MP‐II‐12).

Terminally branched fatty acids are significantly ^13^C‐depleted, with δ^13^C values ranging from −111‰ for *iso*‐C_17_ (MP‐II‐1) to −66‰ for *iso*‐C_16_ (MP‐II‐4). Other branched fatty acids are also ^13^C‐depleted, such as 9me‐C_17_ fatty acid (−101‰ and −88‰ in MP‐II‐13b and MP‐II‐11b, respectively) and, less so, 10me‐C_16_ fatty acid (−57‰ and −51‰ in MP‐II‐11b and MP‐II‐13b, respectively). Other ^13^C‐depleted fatty acids are hydroxy C_16_ fatty acid (δ^13^C = −99‰; MP‐II‐14), hydroxy *iso*‐C_16_ fatty acid (δ^13^C = −94‰; MP‐II‐14), and cyclopropyl C_17:0ω7,8_ fatty acid (δ^13^C = −80‰; MP‐II‐13b). The least ^13^C‐depleted compounds are the saturated *n*‐compounds, with δ^13^C values ranging from −47‰ for *n*‐C_26_ fatty acid (MP‐II‐13b) to −26‰ for *n*‐C_29_ alkane (MP‐II‐13b). A notable deviation from this is both the saturated *n*‐C_23_ fatty acid and its monounsaturated hydrocarbon counterpart, with δ^13^C values ranging from −103‰ to −68‰. Some saturated *n*‐fatty acids are also more ^13^C‐depleted, including C_14_ to C_17_. These *n*‐fatty acids show low values ranging from −85‰ for C_15_ (MP‐II‐14) to −59‰ for C_16_ (MP‐II‐14). The δ^13^C values of *n*‐C_17_ are affected by co‐elution with pristanoic acid (MP‐II‐11b and MP‐II‐13b).

## 5 Discussion

### 5.1 Evidence for an Epigenetic Origin of Native Sulfur and Carbonate at Monte Palco

**FIGURE 7:**
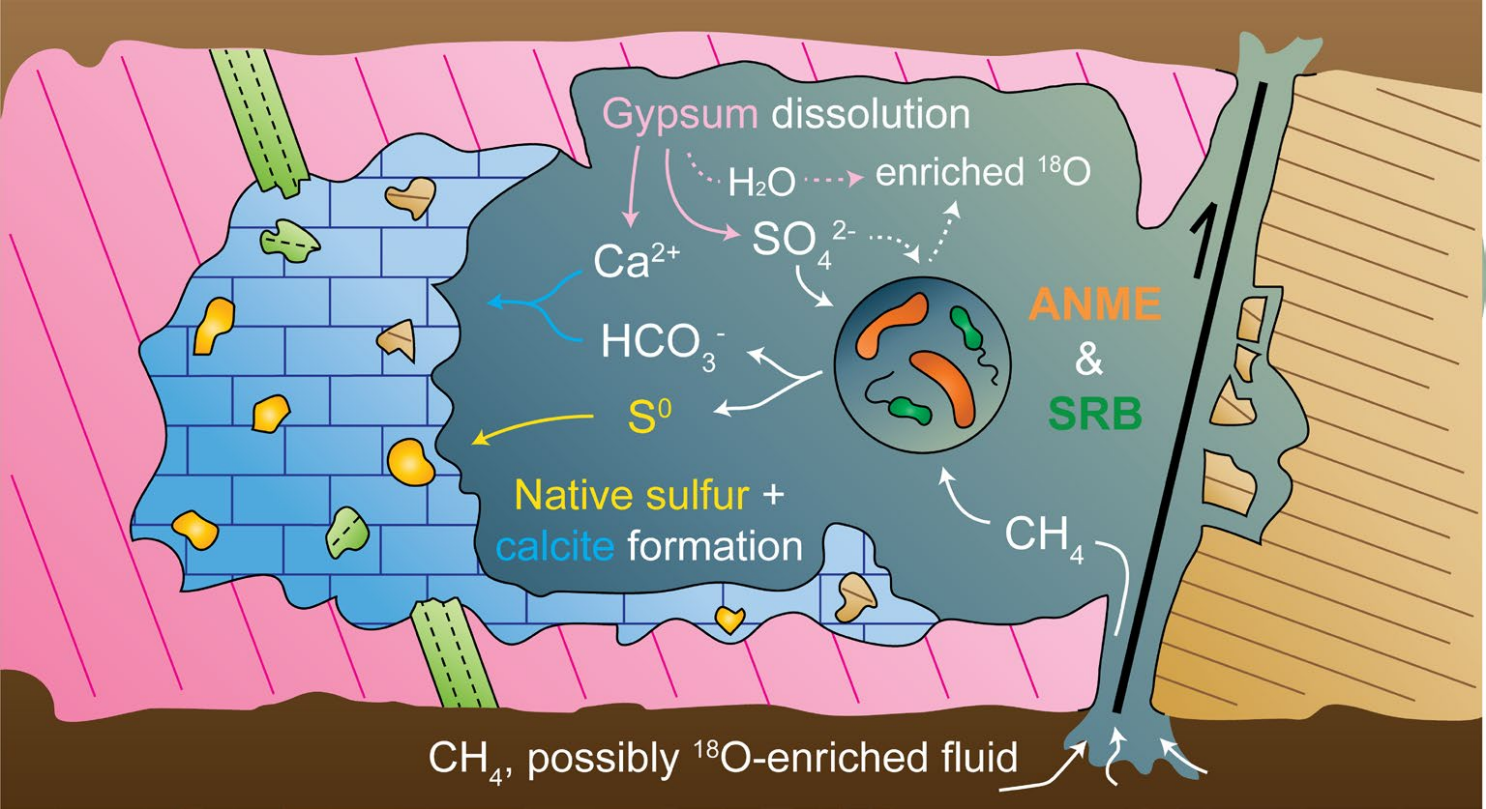
Sketch illustrating the formation of sulfur‐bearing carbonates of the Monte Palco section. Circle is an enlargement showing microbial communities (orange = anaerobic methanotrophic archaea = ANME, green = sulfate‐reducing bacteria = SRB). Lithological units are presented with colors, with blue and gold yellow dots = native sulfur‐bearing carbonate, respectively, pink = gypsum bed of the UG, green = marl of the UG, and light brown = Trubi Formation. The black line and arrow depict a fault.

Field and petrographic evidence for an epigenetic formation of the Monte Palco sulfur‐bearing carbonates is the lack of lamination and sedimentary bedding, much unlike the surrounding sedimentary rocks. Lamination and sedimentary bedding are characteristic of syngenetic sulfur‐bearing carbonates reported from other sites in Sicily (Ziegenbalg et al. 2010) and Spain (Rouwendaal et al. 2023). Microtextures of the Monte Palco sulfur‐bearing carbonates consist mainly of micrite and (micro)sparite. Other microtextures are blocky and partially zoned, scalenohedral calcite crystals, which are commonly related to carbonate formation in meteoric, marine‐phreatic, or shallow burial environments; therefore, agreeing with a subsurface formation (Flügel 2010). The significant spread of δ^13^C_*carbonate*_ values in the various carbonates (from −51.0‰ in the Monte Palco sulfur‐bearing carbonates up to 0.2‰ in UG4 marl) further indicates different phases of carbonate formation (Figure 3a). The sulfur‐bearing carbonates show pronounced ^13^C‐depletion, diagnostic of methane as a major carbon source (cf. Whiticar 1999; Peckmann and Thiel 2004). Isotope measurements of methane in the present Caltanissetta Basin revealed mixed thermogenic and microbial origins with migration of methane from shallow to intermediate depths (Grassa et al. 2004; Madonia et al. 2011; Tassi et al. 2012). The δ^13^C signature of the sulfur‐bearing carbonates is different from that of primary, sedimentary carbonates in the immediate surroundings of the Monte Palco site, which are significantly less ^13^C‐depleted (see Trubi Formation and UG4 marl in Figure 3a). The δ^13^C values of the latter are similar to other late Messinian and early Pliocene carbonates, such as the nearby Calcare di Base carbonates, Upper Gypsum (UG) marlstones, and the foraminifera‐bearing wackestones of the Trubi Formation (Figure 3a). This suggests that the sulfur‐bearing carbonates at Monte Palco precipitated in an environment different from the late Miocene and early Pliocene depositional environments, agreeing with epigenesis. Since some clasts in the sulfur‐bearing carbonates derive from the Pliocene Trubi Formation (Figure 2b), the formation of the sulfur‐bearing carbonate occurred after the deposition of the latter unit and was therefore secondary (Figure 7). A relatively young geological age (< 5.33 Ma; Van Couvering et al. 2000) and a lack of burial agree with the presence of lipids related to secondary mineral formation that suggest relatively minor alteration, such as unsaturated isoprenoids (discussed below) and dominantly ββ hopanoids (cf. Table S2; Seifert and Moldowan 1980).

Additionally, an epigenetic origin of the Monte Palco sulfur‐bearing carbonates is evidenced by the absence of characteristic lipid biomarkers originating from Messinian aquatic organisms, such as phytoplankton (e.g., sterols), anoxygenic phototrophs (e.g., isorenieratane, tetrahymanol), and halophiles (e.g., extended archaeol). Extended archaeol and tetrahymanol are abundant molecular fossils in Messinian gypsum (Natalicchio et al. 2022) and in Messinian non‐sulfur‐bearing carbonates and marls (cf. Birgel et al. 2014; Natalicchio et al. 2017; Sabino et al. 2020, 2021). Similar signatures reflecting Messinian depositional environments were reported for syngenetic sulfur‐bearing carbonates replacing Messinian gypsum elsewhere in Sicily (Ziegenbalg et al. 2012). Interestingly, syngenetic sulfur‐bearing carbonates from the late Miocene succession of the Lorca Basin in Spain also contain abundant water column‐sourced biomarkers (Rouwendaal et al. 2023). Moreover, many of the water column‐derived lipids were preserved as OSCs in the case of the Lorca Basin, reflecting a sulfide‐rich depositional environment. OSCs are found in all sulfur‐bearing carbonates of Monte Palco as well, but none of the samples include any OSCs deriving from phototrophic primary producers. The only OSCs found in the Monte Palco samples are extremely ^13^C‐depleted tail‐to‐tail linked isoprenoids like PMI and squalane, among others.

The oxygen isotope compositions of the micrite and (micro)sparite of the Monte Palco sulfur‐bearing carbonates are characterized by moderate ^18^O‐enrichment (δ^18^O = 2.4‰ to 5.4‰; Figure 3a). An explanation for such ^18^O‐enriched carbonates is precipitation under evaporitic conditions, that is, during gypsum deposition (syngenetic formation), as has been suggested for the nearby Monte Capodarso sulfur‐bearing carbonates (Ziegenbalg et al. 2010). This mechanism is at odds with the interpretation of an epigenetic formation of the Monte Palco sulfur‐bearing carbonates. Alternatively, other processes might have enriched the precipitation fluid in ^18^O, including gas hydrate destabilization (Teichert et al. 2005), clay mineral alteration (Dählmann and De Lange 2003), or the release of connate water from evaporite sediments (Hanor 1994). The existence of gas hydrates has neither been reported or hypothesized for the study area, nor are characteristic petrographic textures associated with gas hydrate destabilization (e.g., Bohrmann et al. 1998; Martire et al. 2010) found in the Monte Palco sulfur‐bearing carbonates. Clay mineral alteration at depth, on the other hand, reputedly produced the unusual high δ^18^O values of methane‐rich fluids that are expulsed by contemporary mud volcanoes in the vicinity of the Monte Palco outcrop (Madonia et al. 2011; Donato et al. 2021). Additionally, it was argued that ^18^O‐enriched authigenic carbonates in the nearby Strait of Sicily precipitated from subsurface brines that were enriched in ^18^O by either Messinian evaporite dissolution or interstitial evaporated seawater from Messinian sediments; these ^18^O‐enriched brines migrated upward through faults (Charlou et al. 2003; Cangemi et al. 2010). Faults directly adjacent to the Monte Palco carbonate outcrops (Figure 1b) could have facilitated the migration of methane and fluids potentially enriched in ^18^O (Figure 7). Fault‐controlled fluid flow in evaporite‐rich basins is a known mechanism to affect surrounding strata, creating reaction zones and promoting fluid and hydrocarbon migration (Bailey et al. 2022; McIntosh et al. 2023). In situ evaporite dissolution is another mechanism to consider for fluid ^18^O‐enrichment, caused by the release of ^18^O‐enriched gypsum hydration water such as found in the gypsum of the vicinal UG units (Aloisi et al. 2022). In an open system, such ^18^O‐enriched fluid contribution from gypsum dissolution water would be flushed out by the in‐ and outflow of external water. In a closed system, this mechanism alone would have a negligible isotopic effect because the dissolution of gypsum would rapidly reach equilibrium concentrations for gypsum saturation in the fluid. However, in a closed system with prominent microbial activity, the oxygen released from sulfate during sulfate reduction, together with the oxygen from gypsum hydration water, could theoretically elevate δ^18^O values when methane and carbonate species resulting from methane oxidation are present (cf. Labrado et al. 2019). Thus, when only a little water is present, this mechanism could potentially impact δ^18^O_*carbonate*_ values. Nevertheless, this mechanism has previously not been reported and thus remains speculative.

Microbial sulfate reduction typically produces ^34^S‐depleted hydrogen sulfide, with a maximum theoretical fractionation of 70‰ (Brunner and Bernasconi 2005; Canfield et al. 2010; Sim et al. 2011), which can further precipitate as native sulfur with negligible effect on ^34^S‐depletion (e.g., Fry et al. 1988; Zerkle et al. 2009; Balci et al. 2012). Native sulfur in the secondary carbonates of Monte Palco is indeed ^34^S‐depleted (δ^34^S = 10.1‰ to 18.9‰) with respect to the original UG sulfate source (average δ^34^S = 22.4‰; García‐Veigas et al. 2018), whereas the residual sulfate (CAS) is significantly ^34^S‐enriched (δ^34^S = 45.1‰ to 61.1‰). However, the ^34^S‐enriched CAS and the relatively small maximum offset (12.3‰) between native sulfur and the original sulfate source suggest a restricted environment, where residual sulfate and native sulfur precipitated with increasingly high δ^34^S values as microbial sulfate consumption was ongoing (the “reservoir effect”; Gomes and Hurtgen 2013, 2015). In other epigenetic deposits, this pattern of increasingly high δ^34^S values was interpreted as evidence for a formation environment characterized by anoxic conditions and negligible fluid flow (i.e., little sulfate removal; Labrado et al. 2019). An adapted Rayleigh equation for the consumption of sulfate by microbial sulfate reduction in a closed system indicates that, for fractionations of both 25‰ (approaching the highest δ^34^S value of native sulfur and lowest δ^34^S value of CAS) and 70‰ (maximum theoretical fractionation of microbial sulfate reduction), a relatively large fraction of sulfate must have been removed upon sulfate conversion to native sulfur at Monte Palco (Figure 3b). Whether the isotope system behaves entirely closed, though, is ambiguous (Labrado et al. 2019); however, an isotopic mass balance approach corroborates that 69%–95% of sulfur released by gypsum dissolution ended up as native sulfur. This high conversion, rather than removal, indicates restricted fluid flow during gypsum dissolution; the supply of solutes like dissolved oxygen must therefore have been limited too. This implies that oxidation of sulfide to native sulfur with oxygen was unlikely and native sulfur formation was probably mediated by microorganisms under anoxic conditions.

### 5.2 Marine Seep‐Like Anaerobic Methanotrophic Archaea (ANME) Mediated Secondary Mineral Formation at Monte Palco

The Monte Palco sulfur‐bearing carbonates contain lipid biomarkers with extremely low δ^13^C values. Similar ^13^C‐depleted biomarkers characterize sulfur‐bearing carbonates from other locations in Sicily (Ziegenbalg et al. 2012). These findings have been interpreted to reflect carbonate precipitation induced by a microbial consortium mediating sulfate‐driven anaerobic oxidation of methane (SD‐AOM) akin to consortia at marine methane seeps (cf. Peckmann and Thiel 2004; Niemann and Elvert 2008; Thiel 2020). Methane seeps, other sulfur‐bearing carbonates from Sicily, and the Monte Palco sulfur‐bearing carbonates not only share abundant ^13^C‐depleted isoprenoids, such as PMI, but also ether‐bound archaeol and *i*GDGTs. Isoprenoids are common membrane constituents of archaea (e.g., Tornabene et al. 1979; Brassell et al. 1981; Chappe et al. 1982; Tourte et al. 2020). Extreme ^13^C‐depletion reflects SD‐AOM and the consequent uptake of the ^13^C‐depleted carbon by archaea for lipid synthesis (Elvert et al. 1999; Hinrichs et al. 1999; Thiel et al. 1999). Different from most seep carbonates, crocetane and *sn2*‐hydroxyarchaeol are absent in the Monte Palco sulfur‐bearing carbonates. Both compounds are biomarkers of ANME‐2 consortia (Niemann and Elvert 2008). ANME‐1 usually produce only minor amounts of *sn2*‐hydroxyarchaeol compared to ANME‐2, but seem to lack crocetane (Hinrichs et al. 1999; Pancost, Hopmans, et al. 2001; Blumenberg et al. 2004). At some methane seeps, *sn2*‐hydroxyarchaeol is accompanied by the less abundant *sn3*‐isomer (Pancost, Hopmans, et al. 2001; Blumenberg et al. 2004; Elvert et al. 2005). Both isomers have been recognized in cultures of mesophilic methanogens (Koga et al. 1993; Sprott et al. 1993). Based on biomarker and genetic evidence, Stadnitskaia et al. (2005) and Elvert et al. (2005) suggested that the source of *sn3*‐hydroxyarchaeol at methane seeps is most likely ANME‐1, but at seeps, this compound was never found as the sole hydroxyarchaeol isomer. Remarkably, the only hydroxyarchaeol isomer found in the Monte Palco sulfur‐bearing carbonates is extremely ^13^C‐depleted *sn3*‐hydroxyarchaeol. The lack of crocetane, the presence of abundant ^13^C‐depleted isoprenoids, and ^13^C‐depleted *sn3*‐hydroxyarchaeol as the sole hydroxyarchaeol isomer have also been reported for syngenetic sulfur‐bearing carbonates from Sicily (Ziegenbalg et al. 2012). Other reports of exclusive *sn3*‐hydroxyarchaeol occurrences related to methane seepage stem from a plumbing network of an Eocene seep system (De Boever et al. 2009) and from fracture‐filling cements of putative gas hydrate‐associated Miocene carbonates (Natalicchio et al. 2012). The Monte Palco biomarker inventory therefore suggests that the archaea involved in carbonate authigenesis after gypsum replacement are closely related to the ANME‐1 cluster.

The otherwise uncommon compound McAr‐2 is present in trace to minor amounts in all Monte Palco sulfur‐bearing carbonates. The only modern seep site where McArs have been found is in a mud breccia from a mud volcano in the Black Sea, where the production of all McArs (acyclic, monocyclic, and bicyclic) has been ascribed to ANME‐1 (Stadnitskaia et al. 2005). Acyclic, monocyclic, and bicyclic McArs with similarly low δ^13^C values have also been found in the previously mentioned Eocene plumbing system (De Boever et al. 2009). Further evidence for the relation of archaea to ANME‐1 at Monte Palco includes the presence of abundant cyclic *i*GDGTs (Figure 5), with GDGT‐2 predominating over GDGT‐0 (Table S2; Blumenberg et al. 2004; Rossel et al. 2008, 2011). Methanogens produce almost exclusively GDGT‐0 in culture, but only rarely produce cyclic *i*GDGTs (Koga et al. 1993; Bauersachs et al. 2015). Ether cleavage of the *i*GDGTs produced acyclic, monocyclic, and bicyclic biphytanes with similar δ^13^C values pointing to SD‐AOM (Figure 6). The GDGTs are accompanied by acyclic, monocyclic, and bicyclic biphytanic diacids, with similar extreme ^13^C‐depletions. Biphytanic diacids have been interpreted as degradation products of *i*GDGTs, although their exact formation mechanism is not well understood (Liu et al. 2016). The uniform δ^13^C values and distribution of ether‐cleaved biphytanes and biphytanic diacids agree with a single source of both compound classes. Similar to the Monte Palco sulfur‐bearing carbonates, other methane‐rich paleoenvironments yielding *sn3*‐hydroxyarchaeol also contain extremely ^13^C‐depleted biphytanic diacids (De Boever et al. 2009). Natalicchio et al. (2012) also reported similarly ^13^C‐depleted acyclic, monocyclic, and bicyclic biphytanes. The compound distribution and the δ^13^C values of the Monte Palco *i*GDGTs and their degradation products further support the interpretation that archaea related to or affiliated with ANME‐1 were present during secondary mineral formation.

The occurrence of the tail‐to‐tail linked C_40_ isoprenoid lycopane in the Monte Palco sulfur‐bearing carbonates is unexpected. Its extreme ^13^C‐depletion is akin to that of the other archaeal biomarkers. This finding implies that lycopane may have derived from the tentative ANME‐1‐like archaea. To date, lycopane has not been described from any marine methane seep environment. It has been assigned to various biological sources in a range of settings. Originally, an archaeal source was put forward (Brassell et al. 1981), but lycopane was later interpreted as a biomarker of unknown marine photoautotrophs from stratified water columns (Wakeham et al. 1993) or the green algae *Botryococcus braunii* race L (Adam et al. 2006). In addition, lycopane was used as a proxy for paleooxicity (Sinninghe Damsté et al. 2003), assuming that unknown photoautotrophs produce lycopane. Interestingly, lycopane is also known as an accessory lipid in the membranes of some extremophilic archaea adapted to high temperatures and pressures (Lattuati et al. 1998; Cario et al. 2015; Salvador‐Castell et al. 2019). The unexpected occurrence of ^13^C‐depleted lycopane in the Monte Palco sulfur‐bearing carbonates, therefore, revived the discussion on the feasibility of an archaeal source of this compound (Rouwendaal et al. 2024).

A key difference of the Monte Palco site with most marine methane seeps is the occurrence of sulfurized archaeal isoprenoid hydrocarbons; recently, sulfurized isoprenoids have been described for the first time in methane‐seep deposits from the Permian of Australia (Wang et al. 2024). Sulfur‐bound isoprenoids show similar distributions and ^13^C‐depletions as the free isoprenoid hydrocarbons (Figures 4a,b and 6). OSCs form as free compounds or macromolecules when reactive organic compounds encounter reduced sulfur species such as hydrogen sulfide (Kutuzov et al. 2020). Reactive compounds were present during carbonate precipitation at Monte Palco, as evidenced by the presence of unsaturated PMIs and squalanes, which have also been found in methane‐seep environments (Elvert et al. 1999, 2001). The production of unsaturated isoprenoid hydrocarbons in archaea has been connected to membrane adaptations in response to environmental conditions such as low temperature, high pressure, high salinity, or possibly as a reversible hydrogen sink to control the internal reduction potential (Tornabene et al. 1978; Nichols et al. 2004; Dawson et al. 2012; Cario et al. 2015).

The potential of sulfurization to preserve information that would otherwise be lost is evidenced by the release of ^13^C‐depleted phytane after desulfurization. Phytane is scarce in the sulfur‐bearing carbonates, if present at all. However, in all samples, its biological precursor occurs as phytanyl chains in archaeol and *sn3*‐hydroxyarchaeol. Phytane released after desulfurization could therefore reflect the preservation of unsaturated archaeols as sulfurized macromolecules, since the δ^13^C value of phytane after desulfurization is similar to that of other isoprenoids. Prominent sulfurization at the Monte Palco site is strikingly exemplified by the presence of ^13^C‐depleted HMO sulfides and the release of HMO after desulfurization. Free HMO is not observed in the Monte Palco sulfur‐bearing carbonates. Both saturated and unsaturated HMO are uncommon in geological samples but have previously been found at hydrothermal vents and in associated sediments of the Guaymas Basin (Holzer et al. 1988), in extremophilic archaea from hydrothermal vents such as the piezo‐hyperthermophilic archaeon *Thermococcus barophilus* (Cario et al. 2015) and the hyperthermophilic methanogen *Methanococcus jannaschii* (Manquin et al. 2004), and at a few deep marine SD‐AOM sites (Stadnitskaia et al. 2005; Bouloubassi et al. 2006; Roberts et al. 2008). In experiments with living ANME‐dominated microbial mats from the Black Sea, free, unsaturated HMOs were found to be associated with ANME‐1 (Bertram et al. 2013). The occurrence of ^13^C‐depleted HMO further supports the notion that archaea involved in epigenetic mineral formation at Monte Palco are likely related to ANME‐1. Consequently, the sulfurization of archaeal lipids during secondary mineral formation reflects sulfidic conditions.

### 5.3 Implications of Compound‐Specific δ^13^C Values of Archaeal Lipids

It is not obvious whether more than one archaeal community was present during secondary mineral formation at Monte Palco. The archaeal lipids show relatively uniform ^13^C‐depletion (δ^13^C_*archaeal*_ = −112‰ ± 7‰; Figure 6), agreeing with a single carbon source and a single or at least strongly dominant group of archaea. However, offsets of more than 10‰ in δ^13^C values between different archaeal lipids exist in the studied samples. For example, the diethers archaeol and *sn3*‐hydroxyarchaeol yielded notably lower δ^13^C values in sample MP‐II‐4 (−113‰ and −120‰, respectively) than in sample MP‐II‐1 (−101‰ and −102‰, respectively). Moreover, isoprenoid diethers are notably abundant in all Monte Palco samples in comparison with GDGTs and their derivatives (Figure 5; Table S2), considering ANME‐1 tend to produce *i*GDGTs rather than diethers at marine seeps (Thiel et al. 2007; Rossel et al. 2008, 2011). GDGTs and their derivatives are also 5‰–16‰ more ^13^C‐depleted than isoprenoid diethers at Monte Palco. Although speculative, these subtle differences in abundances and ^13^C‐depletion could indicate archaeol contributions from a more diverse, unspecified archaeal community besides methanotrophs, as archaeol is a common lipid in various archaea (e.g., Tourte et al. 2020).

Alternatively, the observed variability in δ^13^C values may reflect the response of an ANME‐1‐like community to changing environmental conditions. For example, ANME‐1 synthesize more diethers during their exponential growth, whereas *i*GDGTs are mainly produced during stationary activity (Kellermann et al. 2016). Assuming that methanotrophs were the only archaea present, repeatedly changing growth phases could explain the abundant isoprenoid diethers relative to *i*GDGTs. The pronounced ^13^C‐depletion of GDGTs and their derivatives compared to other lipids can possibly be explained by the observation that both the production of *i*GDGTs and ^13^C uptake into *i*GDGTs are slow in ANME‐1 (Bertram et al. 2013; Kellermann et al. 2016). The least ^13^C‐depleted archaeal lipids are found after desulfurization, including PMI and HMO with respective δ^13^C values of −93‰ and −92‰, which is a ^13^C‐enrichment of +20‰ compared to the average δ^13^C values (see Figure 6). Assuming gradually increasing sulfide concentrations from bacterial sulfate reduction, the sulfurization potential should have increased with time in a restricted environment. A possible explanation for such relative ^13^C‐enrichment is that the pool of methane became less ^13^C‐depleted while the conditions became more sulfidic. Thus, the sulfurized compounds may predominantly record a later stage of microbial processes. The high diether abundances and the differences in lipid δ^13^C values could therefore reflect an adaptation of the methanotrophs to changing environmental conditions.

Another explanation for the observed isotope variability is the known metabolic versatility of ANME‐1, which can perform both SD‐AOM and methanogenesis (e.g., Beulig et al. 2019). Circumstantial evidence of methanogenesis in the Monte Palco carbonate is the presence of HMO sulfides in all samples except MP‐II‐4 and HMO in all desulfurized samples. Experiments with ANME‐1‐dominated enrichment cultures in the absence of methane as a substrate demonstrated that unsaturated HMOs were produced (Bertram et al. 2013). Noticeable ^13^C uptake into HMO from substrates other than methane, especially carbon dioxide, suggested production using another metabolic pathway than SD‐AOM. Interestingly, ANME‐1 has been found to perform both SD‐AOM and autotrophic methanogenesis (Lever et al. 2023). However, it is difficult to discern between SD‐AOM and autotrophic methanogenesis based on ^13^C‐depletion alone because the lipids of autotrophic methanogens can be similarly ^13^C‐depleted as the lipids of ANME‐1 performing SD‐AOM (cf. Londry et al. 2008; Alperin and Hoehler 2009). Because it is impossible to discern between these metabolic pathways based on ^13^C‐depletion alone, the occurrence of methanogenesis in addition to SD‐AOM during secondary mineral formation at Monte Palco, both performed by an ANME‐1‐like community, remains a feasible option to explain the observed subtle heterogeneities in archaeal lipid inventories and ^13^C‐depletions.

### 5.4 Involvement of Sulfate‐Reducing Bacteria in Secondary Mineral Formation

At marine methane seeps, ANME typically perform SD‐AOM in a syntrophic partnership with sulfate‐reducing bacteria of the *Desulfosarcina/Desulfococcus* (*DSS*) group (e.g., Boetius et al. 2000; Michaelis et al. 2002; Elvert et al. 2005). Characteristic bacterial lipids at seeps include ^13^C‐depleted DAGEs (Hinrichs et al. 2000; Pancost, Bouloubassi, et al. 2001; Michaelis et al. 2002; Stadnitskaia et al. 2005). Although these lipids are commonly associated with *DSS* and interpreted as evidence for syntrophy, DAGEs have not been found in cultures of *DSS* to date (Orphan, Hinrichs, et al. 2001; Michaelis et al. 2002; Nauhaus et al. 2007). DAGEs have been observed in other cultures of thermophilic and mesophilic sulfate‐reducing bacteria, commonly in tandem with non‐isoprenoidal monoalkyl glycerol monoethers (Langworthy et al. 1983; Rütters et al. 2001; Grossi et al. 2015; Vinçon‐Laugier et al. 2016); non‐isoprenoidal monoalkyl glycerol monoethers have recently been described as degradation products of mixed acyl‐ether glycerols in *Desulfatibacillum alkenivorans* (Ding et al. 2024). Consequently, DAGEs found at marine seeps are not necessarily sourced from *DSS* (Bouloubassi et al. 2006; Chevalier et al. 2011). DAGEs are not even restricted to sulfate‐reducing bacteria, as these compounds have been observed in cultures of other bacteria as well as in hydrothermal vents, hydrothermal springs, and geothermal sinters (Caillon et al. 1983; Jahnke et al. 2001; Pancost et al. 2005; Sinninghe Damsté et al. 2005; Bradley et al. 2009).

Interestingly, previous biomarker evidence suggested that a seep‐like microbial partnership is also involved in gypsum replacement and resultant carbonate and sulfur formation (Ziegenbalg et al. 2012). ^13^C‐depleted DAGEs are indeed abundant in the Monte Palco sulfur‐bearing carbonates. The apparent absence of monoalkyl glycerol monoethers could be caused by a preservational bias, as DAGEs are less prone to degradation (Vinçon‐Laugier et al. 2018). Yet, the presence of unsaturated isoprenoid hydrocarbons rather suggests excellent preservation, rendering a bias unlikely. The ^13^C‐depleted Monte Palco DAGEs include C_32_ to C_38_ compounds, with the C_33_ DAGEs most abundant, followed by C_34_ and C_32_ or C_35_ compounds (Table S2). The majority of DAGEs have straight and terminally branched alkane chains (usually denoted as “Series I”), which are also common at marine seeps and of which C_33_ (*anteiso*‐C_15_/*anteiso*‐C_15_) DAGE is also typically the most abundant (Pancost, Bouloubassi, et al. 2001; Michaelis et al. 2002; De Boever et al. 2009; Chevalier et al. 2011; Guan et al. 2013, 2018; Kiel et al. 2021; Krake et al. 2022). Many of the same DAGEs are also produced by bacteria co‐occurring with methanogens at sites of active serpentinization (cf. Bradley et al. 2009; Zwicker et al. 2018). The DAGE patterns of the Monte Palco sulfur‐bearing carbonates rather resemble patterns of serpentinization sites, as for example, the C_37_ (10me‐C_16_/10me‐C_16_) DAGE (cf. Bradley et al. 2009). Interestingly, even previously not described DAGEs occur in the Monte Palco sulfur‐bearing carbonates, such as the C_33_ (6me‐C_14_/*iso*‐C_15_) DAGE, DAGEs with dimethyl‐C_15_ chains, and a C_37:2_ (cyclohexyl‐C_11_/cyclohexyl‐C_11_) DAGE (Figure S2). The recognition of these novel DAGEs lends further support to the notion that the Monte Palco paleoenvironment is unlike any of the modern environments shaped by SD‐AOM that have been studied to date.

The observed offset between the δ^13^C_DAGE_ values in the Monte Palco sulfur‐bearing carbonates, spanning from −98‰ to −87‰, is similar to the offset of values reported for some marine seeps (Orphan, House, et al. 2001; Elvert et al. 2003; Wegener et al. 2008; Himmler et al. 2015; Krake et al. 2022) and in other sulfur‐bearing carbonates and subsurface SD‐AOM sites (De Boever et al. 2009; Ziegenbalg et al. 2012; Natalicchio et al. 2012; Drake et al. 2015). The slightly higher relative amounts of DAGEs in Monte Palco samples with high contents of OSCs (Figure 5) support the idea of DAGEs deriving from sulfate‐reducing bacteria. Unlike sulfate‐reducing bacteria from methane‐rich environments, seep‐dwelling, oil‐degrading sulfate‐reducing bacteria have been found to produce overall more DAGEs and DAGEs with longer alkyl chains (C_36_ and C_37_ DAGEs). The larger variety among these chains is accompanied by higher δ^13^C_DAGE_ values compared to methane‐rich environments (Krake et al. 2022). Based on the compound distribution and ^13^C‐depletion, the Monte Palco DAGEs were apparently produced in an environment dominated by SD‐AOM, similar to marine methane seeps and other methane‐rich environments.

The observed dominant chain lengths of DAGEs at Monte Palco suggest moderate temperatures during sulfate reduction because thermophilic sulfate‐reducing bacteria in culture systematically produce longer chains (16 to 17 carbons) than mesophilic sulfate‐reducing bacteria (15 carbons; Vinçon‐Laugier et al. 2017). Additionally, cultures of extremophilic *Aquificales*, grown at temperatures of 85°C, also produce considerably longer chains (18–21 carbons; Jahnke et al. 2001). Although a common source of bacterial lipids from sulfate‐reducing bacteria, based on similar ^13^C‐depletion and distribution patterns, is probable, potential membrane adaptation in response to the environmental conditions during epigenetic gypsum replacement could complicate the assignment of DAGEs to specific producers. In fact, such adaptation likely happened, as evidenced by the relatively abundant cyclic alkanes released after ether cleavage of the DAGEs, including cyclohexyl‐C_11_ (C_17_). Whereas cyclopropyl chains are relatively common in DAGEs (e.g., Pancost, Bouloubassi, et al. 2001; Chevalier et al. 2011; Guan et al. 2013, 2018), cyclohexyl chains are less so. DAGEs with cyclohexyl chains have previously only been reported from marine oil seeps (Krake et al. 2022) and marine mud volcanoes (Pancost, Bouloubassi, et al. 2001; Stadnitskaia et al. 2005; Bouloubassi et al. 2006). The function of cyclization in DAGE chains is unknown, but may represent an adaptation to environmental stress, as suggested for the role of cyclization in other bacterial membrane lipids (De Rosa et al. 1974; Chang and Cronan 1999; Banciu et al. 2005).

The ^13^C‐depleted terminally branched fatty acids found in seep environments are also interpreted as lipids of sulfate‐reducing bacteria (Niemann and Elvert 2008; Thiel 2020). Terminally branched fatty acids are common in a wide range of sulfate‐reducing bacteria (e.g., Ueki and Suto 1979; Taylor and Parkes 1983; Londry et al. 2004; Stackebrandt 2014). Seep‐dwelling sulfate‐reducing bacteria living syntrophic with ANME‐1 are commonly characterized by a high ratio of *anteiso*‐ to *iso*‐C_15_ fatty acids (> 2.0), whereas lower ratios are associated with the syntrophic partners of ANME‐2 (Niemann and Elvert 2008). It has been noted, though, that there is a high overlap in ratios from different communities in seep settings. In the Monte Palco sulfur‐bearing carbonates, this ratio is highly variable, ranging from 0.5 to 1.6, similar to the other SD‐AOM sites (Ziegenbalg et al. 2012; Natalicchio et al. 2012). At methane seeps, such ratios would agree with the presence of sulfate‐reducing bacteria related to ANME‐2 (Elvert et al. 2003; Niemann and Elvert 2008), yet other biomarkers typical of the sulfate reducers associated with ANME‐2, such as ^13^C‐depleted C_16:1ω5_ fatty acid and cyclopropyl‐C_17:0ω5,6_ fatty acid, are missing in the Monte Palco carbonates.

Another characteristic lipid of sulfate‐reducing bacteria, ^13^C‐depleted 10me‐C_16_ fatty acid, is also found in all Monte Palco sulfur‐bearing carbonates. This lipid is produced in large amounts by the genus *Desulfobacter* and in minor amounts by the genus *Desulfovibrio*, rather than the seep‐dwelling, syntrophic *DSS* clade (Dowling et al. 1986; Kohring et al. 1994; Londry et al. 2004). It is less ^13^C‐depleted than other bacterial‐derived lipids, hinting at a sulfate‐reducing bacterial source other than the DAGE‐producing bacteria. Other minor fatty acids are ^13^C‐depleted OH‐ and OH‐*iso*‐C_16_ fatty acids, which have also been reported in several *Desulfovibrio* species (Ueki and Suto 1979; Vainshtein et al. 1992). Cyclopropyl‐C_17:0ω7,8_ fatty acid detected in the sulfur‐bearing carbonates is a prominent lipid in some *Desulfobacter* strains (Taylor and Parkes 1983; Dowling et al. 1986). Further evidence for a diverse community of anaerobic bacteria at Monte Palco is the presence of ^13^C‐depleted 9me‐C_17_ fatty acid, which has previously not been reported as a specific biomarker for sulfate‐reducing bacteria.

Some of the bacteria present during secondary mineral formation also produced hopanoids, as evidenced by their ^13^C‐depletion. Hopanoids are produced by numerous bacteria (Rohmer et al. 1984; Ourisson and Albrecht 1992; Sinninghe Damsté et al. 2004; Cordova‐Gonzalez et al. 2020). These bacterial compounds are also prominent in seep environments and some species of *Desulfovibrio* (Thiel et al. 2003; Blumenberg et al. 2009, 2012). Whether the Monte Palco hopanoids derived from sulfate‐reducing bacteria is ambiguous, as significantly ^13^C‐depleted hopanoids can also be produced by anammox bacteria (Schwartz‐Narbonne et al. 2023). Unsaturated *n*‐C_23_ alkanes (tricos‐10‐ene and 7,14‐tricosadiene) have been suggested to represent biomarkers of bacteria involved in SD‐AOM (Thiel et al. 2001; Michaelis et al. 2002; Peckmann et al. 2007). Their overall abundance and ^13^C‐depletion, along with the release of ^13^C‐depleted *n*‐C_23_ after desulfurization, agree with this concept. Different from other occurrences, *n*‐C_23_ fatty acid and *n*‐C_23_ after ether cleavage have a similar ^13^C‐depletion to the free hydrocarbon counterpart, suggesting degradation of a functionalized compound. Combining genetic and biomarker evidence, Chevalier et al. (2013) suggested the bacterial candidate phylum “Atribacteria” JS1 lineage as a possible producer of *n*‐C_23_ lipids. These anaerobic bacteria are found in the shallow and deep marine subsurface and are capable of the anaerobic degradation of short‐chain *n*‐alkanes while living in syntrophy with formate‐ or hydrogen‐consuming microbes (Webster et al. 2004; Lee et al. 2018; Liu et al. 2019). However, these bacteria are not known as sulfate reducers. Therefore, the *n*‐C_23_ lipids, together with the various bacterial markers and the wide range in bacterial lipid ^13^C‐depletion related to secondary mineral formation (δ^13^C_*bacterial*_ = −92‰ ± 11‰; Figure 6), indicate that several species of sulfate‐reducing bacteria and possibly other bacteria were present during epigenetic mineral formation at Monte Palco.

### 5.5 Native Sulfur Formation as Microbial Response to Environmental Conditions During Gypsum Replacement

The archaeal and bacterial lipid inventories reported here show many similarities with the inventories of the syntrophic SD‐AOM consortiums at marine seeps. The numerous similarities with archaeal biomarkers and ^13^C‐depletion patterns found in other subsurface SD‐AOM sites are also striking. It is likely that the subsurface is a habitat particularly suited either for ANME‐1 or unknown affiliates of ANME‐1. Yet, the Monte Palco archaeal biomarkers also include compounds rarely or not at all reported in other SD‐AOM settings (e.g., lycopane, sulfurized unsaturated C_35_ isoprenoids; Stadnitskaia et al. 2005). These unusual lipid inventories likely result from the particular conditions during gypsum replacement. The environmental conditions during gypsum dissolution either caused ANME‐1 to adapt their lipid inventory (numerous isoprenoid hydrocarbons with 25, 30, 35, and 40 carbons) or were specifically favorable to an unidentified clade related to ANME‐1. Similar arguments apply to the bacterial community, which produced not only cyclic fatty acids and DAGEs with cyclic chains, but also unique lipids such as 9me‐C_17_ fatty acid, various C_23_ compounds, and novel DAGEs.

Gypsum dissolution releases calcium and sulfate ions, and therefore, all microbes presumably had to adapt to increased sulfate concentrations, especially since fluid transport was likely restricted, as indicated by the sulfur isotope data. Moreover, salinity could have potentially been elevated too if fluids derived from or flowed through Messinian sediments, as indicated by the δ^18^O values of carbonate (Charlou et al. 2003; Borin et al. 2009; Cangemi et al. 2010; Madonia et al. 2011; Donato et al. 2021). Incidentally, ANME‐1 are notably better adapted to cope with higher salinities than other methanotrophs (McGenity and Sorokin 2018). In the absence of abundant iron allowing for pyrite formation, sulfide concentrations from bacterial sulfate reduction reached sufficiently high levels to cause sulfurization of organic compounds. Labrado et al. (2019) put forward the concept that a buildup of high concentrations of dissolved sulfate and sulfide is causing microbially mediated native sulfur formation without oxygen. Salt and sulfide stress can cause shifts in the metabolic pathways of microbes, consequently resulting in otherwise uncommon products of microbial processes such as native sulfur. This would in turn explain why native sulfur in the secondary carbonates of Monte Palco precipitated at low fractions of sulfate remaining, that is, at a later stage (Figure 3b). Two of the alternative pathways of sulfur formation suggested by Labrado et al. (2019) involve archaea: (1) ANME performing sulfide oxidation, possibly by shuttling electrons to their sulfate‐reducing bacterial partners, and (2) methanogenesis coupled to sulfide oxidation to native sulfur, possibly in syntrophy with other microbial partners. These two scenarios are supported by observations of ANME generating native sulfur in their cells (Milucka et al. 2012) and possible sulfur cycling by methanogens in a deep‐sea hypersaline basin (Borin et al. 2009). In the Monte Palco carbonates, the uncommon archaeal lipid inventory and the presence of native sulfur apparently characterize the microbial responses to the specific environmental conditions during gypsum dissolution in the subsurface.

Sulfate‐reducing bacteria could play a critical role in native sulfur formation too, either directly through incomplete sulfate reduction or halted acetate metabolism, or as microbial partners of methanotrophic archaea (Labrado et al. 2019). Interestingly, recent findings from pure cultures revealed that several species of sulfate‐reducing bacteria are capable of producing native sulfur directly, with native sulfur contents increasing when salinity increases (Wang et al. 2023). Moreover, a coculture of *Geobacter* and *Desulfovibrio* showed increased sulfate reduction, producing more sulfur than a pure *Desulfovibrio* culture alone, highlighting the potential role of microbial interaction in native sulfur formation (Wang et al. 2023). Geochemical evidence from a syngenetic sulfur‐bearing carbonate also hinted at the role of high salinity and the subsequent adaptation of sulfate‐reducing bacteria in native sulfur formation, but accompanied by dolomite formation in this case—a carbonate mineral commonly forming at higher salinities (Rouwendaal et al. 2023). It remains to be answered whether native sulfur formation at Monte Palco was mediated solely by archaea, solely by sulfate‐reducing bacteria, or by the interaction of different microorganisms (Figure 7). In all instances, sulfur formation seemingly took place in response to high sulfate concentrations, high sulfide levels, and possibly elevated salinity and with ^13^C‐depleted methane as a carbon source.

## 6 Conclusions

Microbial mediation has been suggested as a mechanism for the epigenetic formation of authigenic carbonate and native sulfur after gypsum replacement under anoxic conditions. However, the microbial community participating in this process is poorly constrained. At Monte Palco, Sicily, microbially mediated sulfur‐bearing carbonates were found replacing gypsum of the Messinian Upper Gypsum unit. Indicators of syngenetic formation, such as sedimentary bedding and lamination and water‐column‐derived biomarkers common in other Messinian deposits, are missing in the secondary carbonates. The epigenetic Monte Palco carbonates formed through the microbial oxidation of methane in the subsurface only after the formation of the Pliocene Trubi Formation. Gypsum dissolution was probably sparked by fault‐controlled fluid flow, with carbonates precipitating from methane‐containing fluids, with fluids possibly enriched in ^18^O or ^18^O‐enrichment resulting from low water availability in a closed system with methane and carbon dioxide. However, fluid flow during carbonate and sulfur formation was sluggish because an apparent restriction in sulfate removal resulted in a high degree of conversion of dissolved sulfate to native sulfur. Consequently, any possible solute supply of dissolved oxygen for sulfide oxidation to native sulfur was necessarily limited too, indicating that native sulfur formation occurred under anoxic conditions.

Abundant ^13^C‐depleted lipid biomarkers point to native sulfur and carbonate formation by a community of anaerobic methane‐oxidizing archaea and sulfate‐reducing bacteria performing sulfate‐driven anaerobic oxidation of methane (SD‐AOM). The Monte Palco biomarkers are relatively similar to those of SD‐AOM consortia at marine methane seeps. The biomarker results point toward the involvement of ANME‐1‐like archaea, seep‐like sulfate‐reducing bacteria potentially including species of *Desulfobacter* and *Desulfovibrio*, and other bacterial partners. However, biomarkers extracted from the Monte Palco sulfur‐bearing carbonates previously not reported for ANME and sulfate‐reducing bacteria communities include the C_40_ isoprenoid lycopane, novel DAGEs, and 9me‐C_17_ fatty acid. Such an unusual biomarker inventory demonstrates the dissimilarity of the Monte Palco paleoenvironment from marine methane seeps, with the Monte Palco environment likely characterized by high sulfate concentrations, sulfidic conditions, and possibly elevated salinity. As an adaptation to these environmental stressors, the Monte Palco microbial community mediated carbonate and sulfur formation under anoxic conditions, providing an example that the globally widespread process of native sulfur formation can take place in the absence of molecular oxygen.

## Conflicts of Interest

The authors declare no conflicts of interest.

## Supporting information


Data S1.

## Data Availability

The data that supports the findings of this study are available in the Supporting Information of this article.
