# Supplementary material for: An Anaerobic Microbial Community Mediates Epigenetic Native Sulfur and Carbonate Formation During Replacement of Messinian Gypsum at Monte Palco, Sicily
Source: Geobiology. 2025 Mar 6;23(2):e70015. doi: 10.1111/gbi.70015 (PMC11884234; doi:10.1111/gbi.70015)
Supplement: Supplementary file 1 — Data S1. [file GBI-23-e70015-s001.docx]

***Supplementary Information***

**An anaerobic microbial community mediates epigenetic native sulfur and carbonate formation during replacement of Messinian gypsum at Monte Palco, Sicily**

**Supplement Table S1.** List showing the collected samples, a description of sampled texture and their respective stable isotope values and CAS content.

| **Sample** | **Description textural phase** | **δ^13^C_V-PDB_ (‰)** | **δ^18^O_V-PDB_ (‰)** | **CAS ppm** | **CAS**  **δ^34^S_V-CDT_ (‰)** | **Sulfur**  **δ^34^S_V-CDT_ (‰)** |
| --- | --- | --- | --- | --- | --- | --- |
| Trubi Formation | Foraminifera-bearing wackestone | −1.4 | 1.7 |  |  |  |
| Upper Gypsum cycle 4 (UG4 marl) | Mudstone | 0.2 | −0.4 |  |  |  |
| MP-II-1 | Micrite-sparite matrix | −44.7 | 2.6 |  |  |  |
|  | Micrite-sparite matrix | −45.5 | 3.0 |  |  |  |
| MP-II-4 | Micrite-sparite matrix | −44.1 | 2.5 |  |  |  |
| MP-II-11B | Micrite-sparite matrix | −47.1 | 3.2 | 1243 | 45.1 | 10.1 |
|  | Cavity rim | −43.7 | 2.7 |  |  |  |
|  | Micrite clast | −36.4 | 0.9 |  |  |  |
| MP-II-12 | Micrite-sparite matrix | −40.3 | 2.5 |  |  |  |
|  | Banded sparite cement | −45.7 | 3.3 |  |  |  |
|  | Cavity rim | −40.2 | 2.4 |  |  |  |
| MP-II-13B | Micrite-sparite matrix | −43.3 | 2.6 | 1185 | 51.1 | 12.1 |
|  | Micrite-sparite matrix | −45.1 | 2.8 |  |  |  |
|  | Cavity rim | −43.3 | 2.7 |  |  |  |
| MP-II-14 | Micrite-sparite matrix | −47.1 | 3.2 | 1562 | 50.3 | 18.9 |
|  | Micrite-sparite matrix | −45.6 | 2.9 |  |  |  |
| MP-II-16 | Micrite-sparite matrix | −47.4 | 3.0 | 1272 | 48.2 | 12.8 |
|  | Cavity rim | −44.3 | 3.0 |  |  |  |
| MP-II-17 | Micrite-sparite matrix | −47.8 | 3.3 | 2069 | 55.3 | 11.4 |
|  | Sparite vein | −44.9 | 3.0 |  |  |  |
| MP-I | Micrite-sparite matrix | −51.0 | 5.4 | 2753 | 61.1 | 18.8 |
|  | Cavity rim | −45.2 | 4.0 |  |  |  |

**Supplementary Table S2.** Table containing identified biomarkers in the sulfur-bearing carbonates of Monte Palco, their respective contents in ng/g rock, compound-specific δ^13^C values and relevant standard deviation (σ). Biomarkers are categorized into the fraction the compounds were identified. Summed contents and relative percentages for DAGEs grouped by their relevant carbon size is shown at the bottom of the alcohol fraction tab. Compounds after ether cleavage deriving from DAGEs are highlighted in light purple. Compound abbreviations used are identical to those in the main text. * after the compound name denotes co-elution.

| **DESULFURIZED ASPHALTENES** | | | | | | | | | | | | |
| --- | --- | --- | --- | --- | --- | --- | --- | --- | --- | --- | --- | --- |
| **compound** | **MP-II-1** | | | **MP-II-12** | | | **MP-II-13b** | | | **MP-II-14** | | |
|  | **ng/g rock** | **δ^13^C** | **σ** | **ng/g rock** | **δ^13^C** | **σ** | **ng/g rock** | **δ^13^C** | **σ** | **ng/g rock** | **δ^13^C** | **σ** |
| C15 |  |  |  | 1 |  |  | 1 |  |  | 1 |  |  |
| C16 |  |  |  | 1 |  |  | 1 |  |  | 1 |  |  |
| C17 | trace |  |  | trace |  |  | 1 |  |  | trace |  |  |
| C18 | trace |  |  | 1 |  |  | 1 |  |  | 1 |  |  |
| C19 | trace |  |  | trace |  |  | trace |  |  | trace |  |  |
| C20 | trace |  |  | 1 |  |  | trace |  |  | trace |  |  |
| C21 | trace |  |  | trace |  |  | trace |  |  | trace |  |  |
| C22 | trace |  |  | 1 |  |  | 1 |  |  | trace |  |  |
| C23 | 2 |  |  | 13 | -95 | 0 | 2 | -81 | 0 | 7 | -98 | 0 |
| C24 | 1 |  |  | 1 |  |  | 1 |  |  | trace |  |  |
| C25 | 1 |  |  | 1 |  |  | 1 |  |  | 1 |  |  |
| C26 | 1 |  |  | 1 |  |  | 1 |  |  | 1 |  |  |
| C27 |  |  |  | 1 |  |  | 1 |  |  | trace |  |  |
| C28 | 1 |  |  | 1 |  |  | 1 |  |  | trace |  |  |
| *∑ saturated n-alkanes* | 5 |  |  | 22 |  |  | 11 |  |  | 10 |  |  |
| *iso*-C16 |  |  |  | trace |  |  | trace |  |  | trace |  |  |
| *iso*-C19 |  |  |  | 1 |  |  | 1 |  |  | 1 |  |  |
| *anteiso*-C19 |  |  |  | 1 |  |  | 1 |  |  | 1 |  |  |
| *∑ iso- and anteiso-alkanes* | 0 |  |  | 2 |  |  | 1 |  |  | 2 |  |  |
| phytane | 1 |  |  | 3 |  |  | 2 | -107 | 0 | 3 | -112 | 0 |
| PMI | 1 |  |  | 5 | -102 | 2 | 2 | -93 | 1 | 6 | -111 | 0 |
| squalane | 2 |  |  | 8 | -109 | 1 | 5 | -101 | 0 | 9 | -112 | 0 |
| 2,6,10,14,19,23,27-heptamethyl-octacosane (HMO) | 1 |  |  | 4 |  |  | 2 | -92 | 0 | 3 | -105 | 0 |
| lycopane***** | trace |  |  | 3 |  |  | 1 |  |  | 3 |  |  |
| *∑ saturated acyclic isoprenoids* | 5 |  |  | 22 |  |  | 13 |  |  | 24 |  |  |
| αβ C30 hopane | 1 |  |  | 5 | -86 | 1 | 2 | -86 | 1 | 2 | -89 | 0 |
| ββ C30 hopane | trace |  |  | 1 |  |  | 1 |  |  | 1 |  |  |
| ββ C32 hopane | trace |  |  | trace |  |  | trace |  |  | trace |  |  |
| αβ C34 hopane | trace |  |  | 1 |  |  | trace |  |  | trace |  |  |
| ββ C33 hopane | trace |  |  | 2 |  |  | 1 |  |  | 1 |  |  |
| ββ C34 hopane | 1 |  |  | 13 | -89 | 1 | 6 | -92 | 0 | 6 | -94 | 0 |
| ββ C35 hopane | trace |  |  | 2 |  |  | 2 |  |  | 2 |  |  |
| *∑ hopanoids* | 2 |  |  | 23 |  |  | 11 |  |  | 12 |  |  |
| **∑ compounds after desulfurization of asphaltenes** | **11** |  |  | **69** |  |  | **36** |  |  | **47** |  |  |

| **HYDROCARBONS** | | | | | | | | | | | | | | | | | | |
| --- | --- | --- | --- | --- | --- | --- | --- | --- | --- | --- | --- | --- | --- | --- | --- | --- | --- | --- |
| **compound** | **MP-II-1** | | | **MP-II-4** | | | **MP-II-11b** | | | **MP-II-12** | | | **MP-II-13b** | | | **MP-II-14** | | |
|  | **ng/g rock** | **δ^13^C** | **σ** | **ng/g rock** | **δ^13^C** | **σ** | **ng/g rock** | **δ^13^C** | **σ** | **ng/g rock** | **δ^13^C** | **σ** | **ng/g rock** | **δ^13^C** | **σ** | **ng/g rock** | **δ^13^C** | **σ** |
| C16 |  |  |  | 1 |  |  | 2 |  |  |  |  |  | 3 |  |  | 2 |  |  |
| C17 | 1 |  |  | 3 |  |  | 6 | -33 | 1 | 2 |  |  | 7 | -35 | 2 | 6 | -75 | 3 |
| C18 | 1 |  |  | 5 |  |  | 6 | -30 | 1 | 3 | -35 | 1 | 8 | -32 | 0 | 7 | -45 | 0 |
| C19 | 1 |  |  |  |  |  | 3 | -33 | 2 | 4 | -38 | 1 | 7 | -38 | 0 | 4 | -34 | 2 |
| C20 |  |  |  |  |  |  | 3 | -30 | 2 | 4 | -31 | 0 | 7 | -35 | 1 | 3 |  |  |
| C21 |  |  |  |  |  |  | 2 |  |  | 2 | -46 | 0 | 5 | -42 | 0 |  |  |  |
| C22 |  |  |  |  |  |  | 2 |  |  | 3 | -33 | 1 | 5 |  |  |  |  |  |
| C23 | 14 | -90 | 0 | 3 | -78 | 0 | 18 | -96 | 0 | 22 | -98 | 1 | 18 | -78 | 0 | 65 | -103 | 0 |
| C24 | 1 |  |  | 1 |  |  | 1 |  |  | 1 |  |  | 7 | -33 | 3 | 4 |  |  |
| C25 | 8 |  |  | 2 |  |  | 2 |  |  | 2 |  |  | 7 | -37 | 0 |  |  |  |
| C26 |  |  |  |  |  |  |  |  |  |  |  |  | 7 |  |  | 3 |  |  |
| C27 | 14 | -28 | 0 | 3 | -29 | 0 | 4 |  |  |  |  |  | 10 |  |  | 13 |  |  |
| C28 | 2 |  |  | 1 |  |  | 1 |  |  | 1 |  |  | 8 | -31 | 0 |  |  |  |
| C29 | 8 | -29 | 0 | 5 | -32 | 0 | 7 |  |  |  |  |  | 10 | -26 | 3 | 18 | -28 | 1 |
| C31***** | 4 |  |  | 7 | -35 | 0 | 12 | -43 | 1 |  |  |  | 8 | -44 | 0 | 28 | -39 | 1 |
| C33 |  |  |  | 2 |  |  | 3 |  |  |  |  |  | 3 |  |  |  |  |  |
| *∑ saturated n-alkanes* | 55 |  |  | 33 |  |  | 74 |  |  | 43 |  |  | 118 |  |  | 152 |  |  |
| monounsaturated C23 | 5 |  |  | 2 |  |  | 2 |  |  | 2 | -100 | 0 | 2 |  |  | 8 |  |  |
| monounsaturated C27 | 2 |  |  | 1 |  |  | 2 |  |  | 6 |  |  | trace |  |  | 47 | -37 | 0 |
| pristane | trace |  |  | 4 |  |  |  |  |  |  |  |  |  |  |  |  |  |  |
| phytane | 2 |  |  | 8 |  |  |  |  |  | trace |  |  | trace |  |  | trace |  |  |
| PMI | 56 | -110 | 0 | 11 | -110 | 0 | 27 | -117 | 1 | 59 | -115 | 0 | 54 | -115 | 1 | 135 | -119 | 0 |
| monounsaturated PMI's (PMI:1) | 1 |  |  |  |  |  | 2 |  |  | 1 |  |  | 5 |  |  | 18 |  |  |
| squalane | 37 | -112 | 0 | 17 | -111 | 0 | 69 | -117 | 0 | 72 | -118 | 0 | 74 | -116 | 0 | 115 | -118 | 0 |
| monounsaturated squalane (sq:1) | 1 |  |  | 1 |  |  | 2 |  |  | 3 |  |  | 3 |  |  | 6 |  |  |
| lycopane | 17 | -108 | 0 | 5 | -106 | 0 | 24 | -112 | 1 | 33 | -114 | 0 | 46 | -109 | 1 | 63 | -114 | 0 |
| *∑ saturated acyclic isoprenoids* | 112 |  |  | 33 |  |  | 119 |  |  | 163 |  |  | 174 |  |  | 313 |  |  |
| ∑ PMI sulfides | 22 |  |  | 3 |  |  | 10 |  |  | 11 |  |  | 17 |  |  | 89 |  |  |
| ^(^*^)^ PMI thiane (see Figure S2a) | 10 |  |  | 2 |  |  | 6 |  |  | 6 |  |  | 10 |  |  | 37 | -117 | 1 |
| ∑ squalane sulfides | 8 |  |  | 2 |  |  | 7 |  |  | 12 |  |  | 4 |  |  | 29 |  |  |
| ^(^*^)^ squalane thiolane (see Figure S2b) | 3 |  |  | 2 |  |  | 4 |  |  | 4 | -120 | 3 | 4 |  |  | 15 | -110 | 3 |
| ∑ HMO sulfides | 12 |  |  |  |  |  | 8 |  |  | 11 |  |  | 17 |  |  | 46 |  |  |
| ^(^*^)^ HMO thianes (see Figure S2c) | 3 |  |  | trace |  |  | 4 |  |  | 5 | -107 | 2 | 6 | -103 | 2 | 13 | -114 | 2 |
| lycopane thiane (see Figure S2d) | trace |  |  |  |  |  | 1 |  |  | 3 |  |  | 7 |  |  | 7 |  |  |
| *∑ free acyclic isoprenoid sulfides* | 43 |  |  | 5 |  |  | 26 |  |  | 38 |  |  | 46 |  |  | 172 |  |  |
| hop-17(21)-ene (C30)***** | 2 | -106 | 0 | trace |  |  | 2 |  |  | 3 | -74 | 2 | 2 |  |  | 4 |  |  |
| hop-22(29)-ene (diploptene) (C30) | 2 |  |  | trace |  |  | 1 |  |  | 3 |  |  | 4 |  |  | 8 |  |  |
| hop-21-ene (C30) | 1 |  |  | trace |  |  | 1 |  |  | 1 |  |  | trace |  |  | 4 |  |  |
| C32 thiophene hopane |  |  |  |  |  |  |  |  |  |  |  |  |  |  |  | 21 |  |  |
| C35 thiophene hopane | 4 | -87 |  | trace |  |  | 2 |  |  | 5 |  |  | 5 |  |  | 7 |  |  |
| **∑ hydrocarbons** | **229** |  |  | **87** |  |  | **233** |  |  | **270** |  |  | **359** |  |  | **760** |  |  |

| **ALCOHOLS** | | | | | | | | | | | | | | | | | | |
| --- | --- | --- | --- | --- | --- | --- | --- | --- | --- | --- | --- | --- | --- | --- | --- | --- | --- | --- |
| **compound** | **MP-II-1** | | | **MP-II-4** | | | **MP-II-11b** | | | **MP-II-12** | | | **MP-II-13b** | | | **MP-II-14** | | |
|  | **ng/g rock** | **δ^13^C** | **σ** | **ng/g rock** | **δ^13^C** | **σ** | **ng/g rock** | **δ^13^C** | **σ** | **ng/g rock** | **δ^13^C** | **σ** | **ng/g rock** | **δ^13^C** | **σ** | **ng/g rock** | **δ^13^C** | **σ** |
| C12 |  |  |  |  |  |  | 2 |  |  |  |  |  | 3 |  |  |  |  |  |
| C13 |  |  |  |  |  |  | 1 |  |  |  |  |  | 1 |  |  |  |  |  |
| C14 | 6 |  |  |  |  |  | 8 |  |  | 5 |  |  | 14 |  |  | 1 |  |  |
| C15 | 3 |  |  |  |  |  | 2 |  |  | 5 |  |  | 5 |  |  | 4 |  |  |
| C16 | 19 |  |  | 3 |  |  | 11 |  |  | 12 |  |  | 24 |  |  | 6 |  |  |
| C17 | 8 |  |  | 2 |  |  | 1 |  |  | trace |  |  | 3 |  |  | 4 |  |  |
| C18 | 32 | -29 | 0 | 21 |  |  | 14 |  |  | 10 |  |  | 28 |  |  | 4 |  |  |
| C20 | 7 |  |  | 1 |  |  | 3 |  |  | 3 |  |  | 12 |  |  | trace |  |  |
| C21 |  |  |  |  |  |  | 1 |  |  | trace |  |  | 4 |  |  | trace |  |  |
| C22 | 5 |  |  |  |  |  | 4 |  |  | trace |  |  | 5 |  |  | trace |  |  |
| C23 | trace |  |  |  |  |  | trace |  |  | 1 |  |  | 2 |  |  | trace |  |  |
| C24 | 4 |  |  | 2 |  |  | 2 |  |  | 3 |  |  | 4 |  |  | 1 |  |  |
| C26 | 4 |  |  | 3 |  |  | 5 |  |  | 2 |  |  | 6 |  |  | 5 |  |  |
| C28 | 5 |  |  | 6 |  |  | 3 |  |  | trace |  |  |  |  |  |  |  |  |
| C30 | 3 |  |  | 7 |  |  | 3 |  |  | trace |  |  | 1 |  |  | trace |  |  |
| C31 | trace |  |  |  |  |  | trace |  |  | trace |  |  |  |  |  | trace |  |  |
| C32 | trace |  |  | 1 |  |  | 2 |  |  |  |  |  | 1 |  |  |  |  |  |
| C33 |  |  |  |  |  |  | trace |  |  |  |  |  |  |  |  |  |  |  |
| *∑ saturated n-alcohols* | 95 |  |  | 47 |  |  | 63 |  |  | 40 |  |  | 113 |  |  | 26 |  |  |
| phytanol | 32 | -102 | 0 | 3 |  |  | 14 |  |  | 2 |  |  | 33 |  |  | 16 |  |  |
| *sn2*-phytanyl glycerol monoether | 5 |  |  | 2 |  |  | 3 |  |  | 2 |  |  | 4 |  |  | 2 |  |  |
| *sn3*-phytanyl glycerol monoether | trace |  |  | trace |  |  | 1 |  |  | 8 |  |  | 2 |  |  | 3 |  |  |
| archaeol | 129 | -102 | 0 | 33 | -113 | 0 | 78 |  |  | 201 |  |  | 66 |  |  | 226 |  |  |
| *sn3*-hydroxyarchaeol | 69 | -101 | 0 | 14 | -120 | 0 | 30 |  |  | 114 |  |  | 63 |  |  | 149 |  |  |
| bicyclic McAr | trace |  |  | trace |  |  | trace |  |  | trace |  |  | 7 |  |  | trace |  |  |
| *∑ isoprenoid alcohols* | 235 |  |  | 52 |  |  | 126 |  |  | 328 |  |  | 176 |  |  | 396 |  |  |
| C32 (*iso*-C14/*anteiso*-C15) DAGE |  |  |  |  |  |  | 6 |  |  | 13 |  |  | trace |  |  | 14 |  |  |
| C32 (*n*-C14/*anteiso*-C15) DAGE | 32 | -90 | 0 | 2 |  |  | 14 |  |  | 73 |  |  | 25 |  |  | 54 |  |  |
| C33 (6me-C14/*iso*-C15) DAGE ***** | 13 | -94 | 0 | trace |  |  | 12 |  |  | 35 |  |  | 13 |  |  | 32 |  |  |
| C33 (*iso*-C15/*iso*-C15) DAGE ***** | 9 |  |  | trace |  |  | 4 |  |  | 14 |  |  | 12 |  |  | 12 |  |  |
| C33 (*iso*-C15/*anteiso*-C15) DAGE | 25 | -98 | 0 | trace |  |  | 8 |  |  | 45 |  |  | 16 |  |  | 33 |  |  |
| C33 (*anteiso*-C15/*anteiso*-C15) DAGE | 130 | -95 | 0 | 7 |  |  | 30 |  |  | 210 |  |  | 68 |  |  | 146 |  |  |
| C33 (*n*-C15/*n*-C15) DAGE  + C33 (*n*-C16/*n*-C14) DAGE * |  |  |  |  |  |  | 3 |  |  | 5 |  |  | 6 |  |  | trace |  |  |
| C34 (*iso*-C16/*anteiso*-C15) DAGE  + C34 (dimethyl-C15/*n*-C14) DAGE * | 25 | -95 | 0 | 4 |  |  | 13 |  |  | 60 |  |  | 29 |  |  | 38 |  |  |
| C34 DAGE (*n*-C16/*anteiso*-C15) DAGE | 24 | -92 | 0 | 4 |  |  | 16 |  |  | 63 |  |  | 23 |  |  | 53 |  |  |
| C35 (dimethyl-C15/*iso*-C15) DAGE |  |  |  | trace |  |  | 8 |  |  | trace |  |  | 21 |  |  |  |  |  |
| C35 (*iso*-C16/*iso*-C16) DAGE  + C35 (dimethyl-C15/*anteiso*-C15) DAGE * |  |  |  |  |  |  |  |  |  | 6 |  |  | 11 |  |  |  |  |  |
| C35 (10me-C16/*anteiso*-C15) DAGE | 8 | -88 | 0 |  |  |  | 9 |  |  | 25 |  |  | 10 |  |  | 20 |  |  |
| C35 (*iso*-C16/*n*-C16) DAGE  + C35 (*iso*-C17/*anteiso*-C15) DAGE * | 8 |  |  | trace |  |  | 5 |  |  | 11 |  |  | 12 |  |  | 11 |  |  |
| C35 (*anteiso*-C17/*anteiso*-C15) DAGE | 17 | -87 | 0 |  |  |  | 4 |  |  | 26 |  |  | 8 |  |  | 17 |  |  |
| C35 (*n*-C16/*n*-C16) DAGE | trace |  |  |  |  |  | 4 |  |  | 10 |  |  | 22 |  |  | 14 |  |  |
| C36:1 (10Me-C16/cp-C16) DAGE | trace |  |  |  |  |  | 3 |  |  | 4 |  |  | 8 |  |  | trace |  |  |
| C37 (10me-C16/10me-C16) DAGE | trace |  |  |  |  |  | 3 |  |  | 5 |  |  | 8 |  |  | trace |  |  |
| C36:1 (?/?) DAGE | trace |  |  |  |  |  | 5 |  |  | 12 |  |  | 24 |  |  | 14 |  |  |
| C37:1 (?/?) DAGE |  |  |  |  |  |  |  |  |  |  |  |  | 7 |  |  |  |  |  |
| C38:1 (*n*-16/cp-19) DAGE |  |  |  |  |  |  |  |  |  | 9 |  |  | 16 |  |  | 20 |  |  |
| C37:2 (ch-C17:1/ch-C17:1) DAGE * | 15 |  |  | 3 |  |  | 7 |  |  | 11 |  |  | 24 |  |  | 14 |  |  |
| *∑ dialkyl glycerol diethers (DAGEs)* | 306 |  |  | 20 |  |  | 152 |  |  | 637 |  |  | 362 |  |  | 492 |  |  |
| cholesterol | 18 | -26 | 0 | 3 |  |  | 3 |  |  | 4 |  |  | 14 |  |  | 52 |  |  |
| β-sitosterol | 27 | -35 | 0 | 7 |  |  |  |  |  |  |  |  | 43 |  |  |  |  |  |
| **∑ alcohols** | **680** |  |  | **128** |  |  | **344** |  |  | **1009** |  |  | **707** |  |  | **966** |  |  |

| **Total carbons DAGEs** | **MP-II-1** | | **MP-II-4** | | **MP-II-11b** | | **MP-II-12** | | **MP-II-13b** | | **MP-II-14** | |
| --- | --- | --- | --- | --- | --- | --- | --- | --- | --- | --- | --- | --- |
|  | **ng/g rock** | **rel. %** | **ng/g rock** | **rel. %** | **ng/g rock** | **rel. %** | **ng/g rock** | **rel. %** | **ng/g rock** | **rel. %** | **ng/g rock** | **rel. %** |
| **∑ C32** | 32 | 11% | 2 | 13% | 19 | 13% | 86 | 14% | 25 | 7% | 68 | 14% |
| **∑ C33** | 177 | 58% | 7 | 34% | 56 | 37% | 308 | 48% | 115 | 32% | 224 | 45% |
| **∑ C34** | 49 | 16% | 8 | 40% | 29 | 19% | 123 | 19% | 52 | 14% | 91 | 19% |
| **∑ C35** | 33 | 11% | 0 | 0% | 30 | 19% | 78 | 12% | 83 | 23% | 61 | 12% |
| **∑ C36** | 0 | 0% | 0 | 0% | 8 | 5% | 16 | 3% | 32 | 9% | 14 | 3% |
| **∑ C37** | 15 | 5% | 3 | 13% | 9 | 6% | 16 | 3% | 38 | 11% | 14 | 3% |
| **∑ C38** | 0 | 0% | 0 | 0% | 0 | 0% | 9 | 1% | 16 | 5% | 20 | 4% |

| **GDGTs** | | | | | | | | | | | | |
| --- | --- | --- | --- | --- | --- | --- | --- | --- | --- | --- | --- | --- |
| **compound** | **MP-II-1** | | **MP-II-4** | | **MP-II-11b** | | **MP-II-12** | | **MP-II-13b** | | **MP-II-14** | |
|  | **ng/g rock** | **rel. %** | **ng/g rock** | **rel. %** | **ng/g rock** | **rel. %** | **ng/g rock** | **rel. %** | **ng/g rock** | **rel. %** | **ng/g rock** | **rel. %** |
| GDGT-0 | 10 | 22% | 3 | 30% | 16 | 31% | 37 | 19% | 24 | 27% | 27 | 23% |
| GDGT-1 | 8 | 18% | 2 | 21% | 9 | 17% | 37 | 19% | 14 | 16% | 19 | 16% |
| GDGT-2 | 15 | 33% | 2 | 22% | 13 | 25% | 70 | 36% | 23 | 26% | 38 | 32% |
| GDGT-3 | 9 | 20% | 2 | 18% | 8 | 16% | 31 | 16% | 15 | 17% | 23 | 19% |
| GDGT-4 | 3 | 7% | 1 | 9% | 4 | 8% | 13 | 7% | 10 | 11% | 9 | 8% |
| crenarchaeol | traces |  | traces |  | 2 | 4% | 4 | 2% | 4 | 4% | 3 | 3% |
| crenarchaeol regioisomer | traces |  | traces |  | traces |  | traces |  | traces |  | 1 | 0% |
| **∑ GDGTs** | 46 |  | 11 |  | 52 |  | 192 |  | 90 |  | 120 |  |

| **ETHER CLEAVAGE** | | | | | | | | | |
| --- | --- | --- | --- | --- | --- | --- | --- | --- | --- |
| **compound** | **MP-II-12** | | | **MP-II-13b** | | | **MP-II-14** | | |
|  | **ng/g rock** | **δ^13^C** | **σ** | **ng/g rock** | **δ^13^C** | **σ** | **ng/g rock** | **δ^13^C** | **σ** |
| C14 | 21 | -93 | 0 |  |  |  | 13 | -93 | 2 |
| C15 | 6 |  |  |  |  |  | 5 |  |  |
| C16 | 59 | -97 | 0 | 21 |  |  | 38 | -99 | 1 |
| C17 | 3 |  |  | 7 |  |  | 3 |  |  |
| C20 | 4 |  |  | 21 | -28 | 1 | 3 |  |  |
| C21 | 1 |  |  | 9 |  |  | 1 |  |  |
| C22 | 2 |  |  | 14 | -31 | 1 | 2 |  |  |
| C23 | 6 | -94 | 2 | 6 |  |  | 7 | -97 | 0 |
| C24 | 3 |  |  | 9 | -37 | 3 | 3 |  |  |
| C25 |  |  |  | 6 |  |  |  |  |  |
| C26 | 2 |  |  | 7 |  |  | 6 | -48 | 0 |
| C27 |  |  |  | 15 | -35 | 1 |  |  |  |
| C28 | 1 |  |  | 9 | -36 | 2 | 6 | -50 | 0 |
| C29 |  |  |  | 18 | -31 | 1 |  |  |  |
| C30 |  |  |  | 8 | -37 | 1 | 3 |  |  |
| C31 |  |  |  | 20 | -36 | 0 |  |  |  |
| C32 |  |  |  | 5 |  |  |  |  |  |
| C33 |  |  |  | 8 | -36 | 0 |  |  |  |
| *∑ saturated n-alkanes* | 108 |  |  | 182 |  |  | 88 |  |  |
| *iso*-C14 | 2 |  |  |  |  |  | 2 |  |  |
| *iso*-C15 | 28 | -107 | 1 |  |  |  | 17 | -107 | 1 |
| *anteiso*-C15 | 207 | -101 | 1 |  |  |  | 99 | -102 | 0 |
| *iso*-C16 | 27 | -101 | 0 | 2 |  |  | 12 | -102 | 0 |
| *iso*-C17 | 8 | -107 | 0 | 4 |  |  | 4 |  |  |
| *anteiso*-C17 | 13 | -95 | 0 | 4 |  |  | 5 |  |  |
| *iso*-C18 | 2 |  |  |  |  |  | 1 |  |  |
| *iso*-C19 | 1 |  |  | 1 |  |  | 1 |  |  |
| *anteiso*-C19 | 1 |  |  | 3 |  |  | 1 |  |  |
| 6me-C14 | 10 | -95 | 1 |  |  |  | 5 | -98 | 0 |
| 5me-C14 | 2 |  |  |  |  |  | 1 |  |  |
| 4me- and 5me-C15 (likely derived from cyclopropyl C16)***** | 3 |  |  |  |  |  | 3 |  |  |
| 7me- and 8me-C18 (likely derived from cyclopropyl C19)* | 2 |  |  | 4 |  |  | 2 |  |  |
| 2,7-dimethyl-C15 | 6 | -98 | 1 |  |  |  | 3 |  |  |
| 10Me-C16 | 25 | -89 | 0 | 12 |  |  | 12 | -94 | 1 |
| *∑ methyl alkanes* | 337 |  |  | 30 |  |  | 168 |  |  |
| cyclohexyl-C11 (C17) | 34 | -92 | 1 | 14 |  |  | 19 | -89 | 0 |
| cyclohexyl-C12 (C18) | trace |  |  |  |  |  | trace |  |  |
| cyclohexyl-C14 (C20) | 3 |  |  | 6 |  |  | 3 |  |  |
| *∑ cyclohexyl alkanes* | 37 |  |  | 19 |  |  | 22 |  |  |
| phytane | 279 | -107 | 1 | 113 | -106 | 1 | 154 | -107 | 1 |
| acyclic biphytane | 62 | -115 | 0 | 36 | -111 | 0 | 44 | -114 | 2 |
| monocyclic biphytane | 63 | -117 | 1 | 39 | -115 | 0 | 47 | -116 | 0 |
| bicyclic biphytane | 49 | -119 | 0 | 31 | -116 | 0 | 36 | -117 | 1 |
| *∑ (bi)phytanes* | 453 |  |  | 219 |  |  | 281 |  |  |
| ββ C34 homohopane | 3 |  |  | trace |  |  | 4 |  |  |
| **∑ compounds after ether-cleavage of alcohol fraction** | **939** |  |  | **450** |  |  | **562** |  |  |
| **∑ DAGE-derived products** | **ng/g rock** |  |  | **ng/g rock** |  |  | **ng/g rock** |  |  |
|  | 454 |  |  | 68 |  |  | 241 |  |  |

| **FATTY ACIDS** | | | | | | | | | | | | | | | | | | |
| --- | --- | --- | --- | --- | --- | --- | --- | --- | --- | --- | --- | --- | --- | --- | --- | --- | --- | --- |
| **compound** | **MP-II-1** | | | **MP-II-4** | | | **MP-II-11b** | | | **MP-II-12** | | | **MP-II-13b** | | | **MP-II-14** | | |
|  | **ng/g rock** | **δ^13^C** | **σ** | **ng/g rock** | **δ^13^C** | **σ** | **ng/g rock** | **δ^13^C** | **σ** | **ng/g rock** | **δ^13^C** | **σ** | **ng/g rock** | **δ^13^C** | **σ** | **ng/g rock** | **δ^13^C** | **σ** |
| C13 |  |  |  | 1 |  |  | 3 |  |  |  |  |  |  |  |  | 3 |  |  |
| C14 | 30 |  |  | 14 |  |  | 33 | -51 | 0 | 4 |  |  | 9 | -53 | 0 | 27 | -78 | 0 |
| C15 | 29 |  |  | 9 |  |  | 26 | -51 | 0 | 4 |  |  | 19 | -72 | 1 | 29 | -85 | 1 |
| C16 | 186 | -47 | 0 | 99 | -33 | 0 | 356 | -34 | 0 | 112 |  |  | 227 | -43 | 1 | 117 | -59 | 0 |
| C17***** | 12 |  |  | 9 |  |  | 9 | -43 | 1 | 5 |  |  | 9 | -60 | 0 | 7 | -68 | 1 |
| C18 | 65 | -33 | 0 | 34 | -25 | 0 | 243 | -32 | 0 | 118 |  |  | 216 | -33 | 1 | 51 | -37 | 1 |
| C19 | 4 |  |  | 4 |  |  | 3 |  |  | trace |  |  | 5 |  |  | 4 |  |  |
| C20 | 28 |  |  | 8 |  |  | 9 | -31 | 0 | 2 |  |  | 5 | -27 | 2 | 7 | -44 | 2 |
| C21 |  |  |  | 3 |  |  | 3 |  |  |  |  |  | 4 | -31 | 3 | 4 |  |  |
| C22 | 27 |  |  | 10 |  |  | 9 | -28 | 2 | 3 |  |  | 7 | -31 | 1 | 7 | -31 | 3 |
| C23 | 5 |  |  | 5 |  |  | 4 |  |  |  |  |  | 3 | -68 | 1 | 4 |  |  |
| C24 | 15 |  |  | 20 |  |  | 19 | -36 | 1 |  |  |  | 15 | -55 | 1 | 11 | -39 | 2 |
| C25***** | trace |  |  | 5 |  |  | 7 | -60 | 0 |  |  |  | 10 | -85 | 2 | 5 |  |  |
| C26 | 12 |  |  | 16 |  |  | 20 | -42 | 0 |  |  |  | 15 | -47 | 0 | 14 | -45 | 2 |
| C27 |  |  |  | 2 |  |  | 3 |  |  | 2 |  |  | trace |  |  | 4 |  |  |
| C28 | 9 |  |  | 6 |  |  | 8 | -30 | 0 |  |  |  | 8 | -38 | 1 | 13 | -41 | 1 |
| C29 |  |  |  | 1 |  |  | 2 |  |  |  |  |  | trace |  |  | 4 |  |  |
| C30 | 6 |  |  | 3 |  |  | 5 |  |  |  |  |  | 3 | -27 | 1 | 7 | -32 | 1 |
| *∑ saturated  n-fatty acids* | 428 |  |  | 250 |  |  | 760 |  |  | 251 |  |  | 555 |  |  | 316 |  |  |
| C16:1 | 53 |  |  | 11 |  |  | 14 | -19 | 0 | 8 |  |  | 17 | -23 | 2 |  |  |  |
| C18:2***** | co-elution |  |  | 185 |  |  | 20 |  |  | 20 |  |  | 53 | -35 | 1 | 12 |  |  |
| C18:1***** | 149 |  |  | 17 |  |  | 64 | -31 | 1 | 6 |  |  | 15 | -42 | 0 | 12 | -55 | 1 |
| *∑ unsaturated  n-fatty acids* | 202 |  |  | 212 |  |  | 98 |  |  | 34 |  |  | 86 |  |  | 23 |  |  |
| *iso*-C13 |  |  |  | 1 |  |  | 2 |  |  |  |  |  |  |  |  | 2 |  |  |
| *anteiso*-C13 |  |  |  | 1 |  |  | 1 |  |  |  |  |  |  |  |  | 1 |  |  |
| *iso*-C14 | 8 |  |  | 3 |  |  | 7 | -102 | 2 | 1 |  |  | 2 |  |  | 6 | -102 | 0 |
| *anteiso*-C14 |  |  |  |  |  |  | 3 |  |  | trace |  |  |  |  |  | 2 |  |  |
| *iso*-C15 | 19 | -82 | 0 | 12 | -86 | 0 | 30 | -98 | 0 | 5 |  |  | 11 | -92 | 0 | 25 | -106 | 0 |
| *anteiso*-C15 | 31 | -77 | 0 | 6 | -72 | 0 | 19 | -88 | 1 | 6 |  |  | 10 | -85 | 1 | 23 | -97 | 0 |
| *iso*-C16 | 27 | -69 | 0 | 15 | -66 | 0 | 15 | -90 | 1 | 4 |  |  | 17 | -86 | 0 | 16 | -101 | 1 |
| *iso*-C17 | 17 | -111 | 0 | 9 | -103 | 0 | 27 | -108 | 1 | 6 |  |  | 28 | -106 | 1 | 23 | -110 | 0 |
| *anteiso*-C17 | 28 | -96 | 0 | 4 | -81 | 0 | 13 | -94 | 1 | 10 |  |  | 4 | -92 | 0 | 19 | -101 | 0 |
| *iso*-C19 | 4 |  |  |  |  |  | 3 |  |  | 2 |  |  | 5 |  |  | 4 |  |  |
| *anteiso*-C19 | 5 |  |  | 3 |  |  | 3 |  |  | 2 |  |  | 5 |  |  | 5 | -103 | 1 |
| *∑ iso- and anteiso-fatty acids* | 138 |  |  | 54 |  |  | 123 |  |  | 36 |  |  | 82 |  |  | 125 |  |  |
| hydroxy *iso*-C16 |  |  |  | 3 |  |  | 4 |  |  |  |  |  |  |  |  | 7 | -94 | 1 |
| cyclopropyl C17:0ω7,8 | 13 |  |  | 8 |  |  | 3 |  |  | trace |  |  | 11 | -80 | 1 | trace |  |  |
| cyclohexyl+C11 (C17) |  |  |  |  |  |  |  |  |  |  |  |  | co-elution |  |  | 9 | -74 | 2 |
| cyclopropyl C19:0 | 3 |  |  | 2 |  |  | 2 |  |  | 2 |  |  | 5 |  |  | 4 |  |  |
| 10me-C16 | 16 |  |  | 4 |  |  | 13 | -57 | 1 | 5 |  |  | 15 | -51 | 1 | 4 |  |  |
| 12me-C16 | 15 |  |  | 3 |  |  |  |  |  | 3 |  |  | 13 | -37 | 0 |  |  |  |
| 9me-C17 | 5 |  |  |  |  |  | 4 | -88 | 2 | 5 |  |  | 7 | -101 | 1 | trace |  |  |
| 14me-C17 | 3 |  |  | 2 |  |  |  |  |  |  |  |  | 10 | -32 | 0 | 4 | -50 | 1 |
| 10me-C18 |  |  |  | 5 |  |  |  |  |  |  |  |  | 4 | -35 | 0 | 12 |  |  |
| 17me-C24 |  |  |  |  |  |  |  |  |  | 3 |  |  | 3 |  |  |  |  |  |
| 21me-C26 |  |  |  |  |  |  | 4 |  |  |  |  |  | 5 | -106 | 3 | 4 |  |  |
| *∑ other branched and  cyclic fatty acids* | 54 |  |  | 27 |  |  | 30 |  |  | 19 |  |  | 73 |  |  | 42 |  |  |
| C30 diacid | 27 |  |  |  |  |  | 20 | -104 | 0 |  |  |  |  |  |  | 15 | -108 | 0 |
| C31 diacid | 25 |  |  |  |  |  | 13 | -101 | 1 | 2 |  |  |  |  |  | 11 |  |  |
| C32 diacid | 33 |  |  |  |  |  | 11 | -108 | 2 |  |  |  | 3 |  |  | 9 | -102 | 0 |
| *∑ diabolic acids* | 84 |  |  | 0 |  |  | 44 |  |  | 2 |  |  | 3 |  |  | 34 |  |  |
| C29 28-norhopanoic |  |  |  |  |  |  |  |  |  | 8 |  |  |  |  |  |  |  |  |
| C31 28-norhopanoic | trace |  |  |  |  |  | 5 |  |  | 5 |  |  | 28 | -91 | 0 | 10 | -93 | 2 |
| αβ C32 homohopanoic | trace |  |  |  |  |  | 8 | -97 | 3 | 3 |  |  | 8 | -92 | 2 | 6 | -93 | 3 |
| ββ C31 homohopanoic |  |  |  |  |  |  |  |  |  |  |  |  | 8 |  |  | 13 |  |  |
| ββ C32 homohopanoic | 165 | -94 | 0 | 47 | -88 | 0 | 255 | -96 | 0 | 15 |  |  | 271 | -92 | 0 | 116 | -95 | 0 |
| ββ C33 homohopanoic |  |  |  |  |  |  | 4 |  |  | 3 |  |  | 9 | -92 | 0 | 4 |  |  |
| ββ C34 homohopanoic | 13 |  |  |  |  |  | 8 |  |  | 8 |  |  | 21 | -93 | 0 | 12 | -91 | 0 |
| *∑ hopanoic acids* | 178 |  |  | 47 |  |  | 279 |  |  | 43 |  |  | 345 |  |  | 161 |  |  |
| pristanoic acid* | 5 |  |  |  |  |  | 2 |  |  | co-elution |  |  | 9 |  |  |  |  |  |
| phytanic acid* | 52 |  |  | 5 |  |  | 4 | -73 | 4 | 46 |  |  | 47 | -73 | 0 | 25 | -105 | 1 |
| acyclic biphytanic diacid | 98 | -115 | 0 | 5 | -125 | 0 | 57 | -114 | 0 | 57 |  |  | 115 | -112 | 0 | 50 | -116 | 0 |
| monocyclic biphytanic diacid | 120 | -115 | 0 | 5 | -124 | 0 | 63 | -116 | 0 | 16 |  |  | 107 | -114 | 0 | 52 | -116 | 1 |
| bicyclic biphytanic diacid | 93 | -117 | 0 | trace | -126 | 0 | 52 | -115 | 0 | 6 |  |  | 81 | -113 | 0 | 38 | -116 | 0 |
| *∑ isoprenoid acids* | 368 |  |  | 16 |  |  | 178 |  |  | 124 |  |  | 359 |  |  | 165 |  |  |
| hydroxy C16 acid | 25 |  |  | 4 |  |  | 4 |  |  |  |  |  |  |  |  | 30 | -99 | 3 |
| **∑ fatty acids** | **1477** |  |  | **611** |  |  | **1515** |  |  | **508** |  |  | **1503** |  |  | **898** |  |  |

**
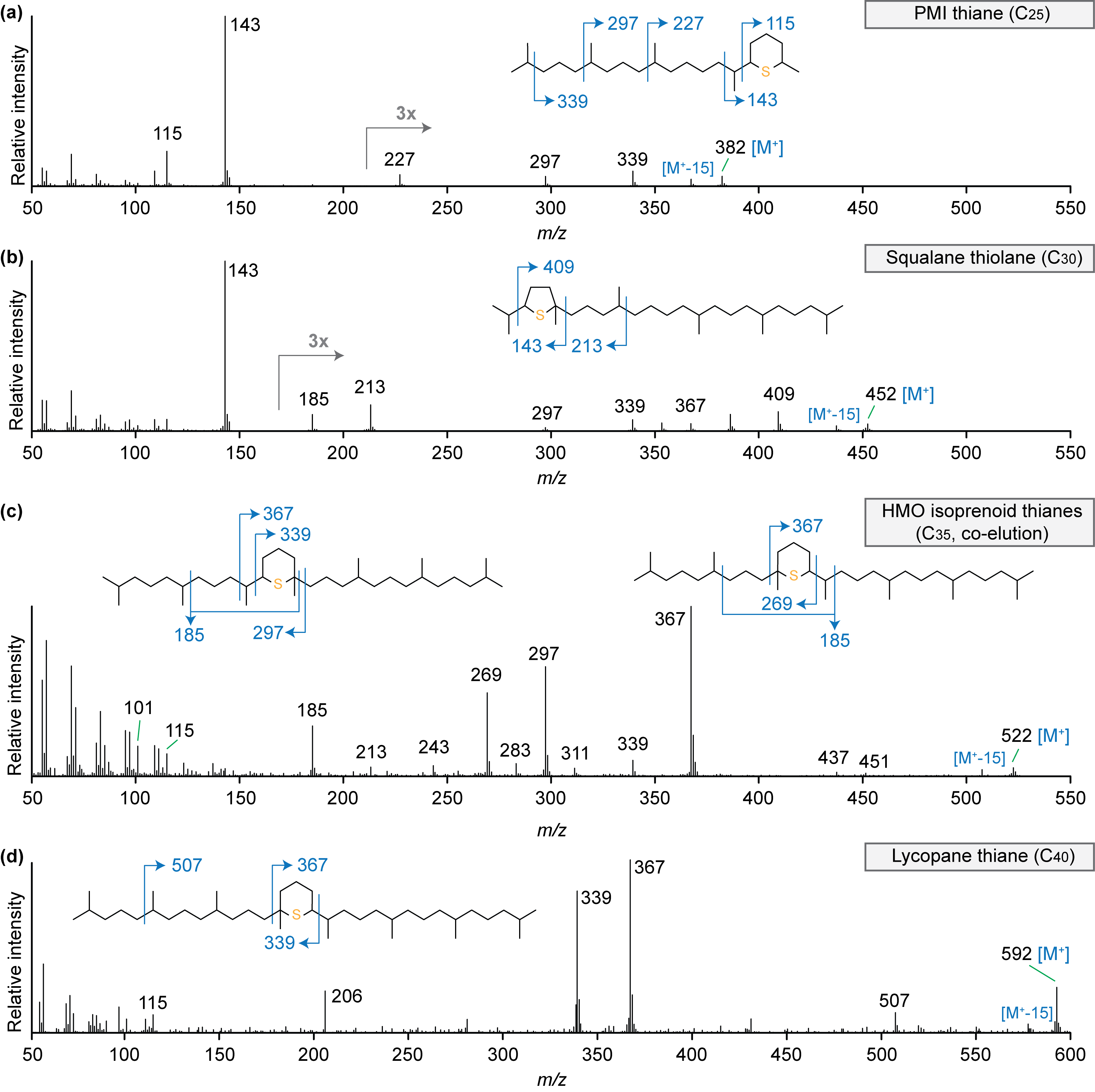
**

**Figure S1:** Mass spectra and structures of selected organic sulfur-containing isoprenoids. Arrows and numbers in blue show relevant mass fragmentation of the compounds. S = sulfur, [M+] = molecular ion. Compounds shown are (a) PMI thiane, (b) squalane thiolane, (c) two co-eluting HMO isoprenoid thianes and (d) lycopane thiane. The tentative interpretation and assignment of the mass spectra is based on both mass spectra for squalane-derived and lycopane-derived sulfides (cf. Grice et al., 1998; Wu et al., 2020), as well as the desulfurized asphaltenes containing their hydrocarbon counterparts.


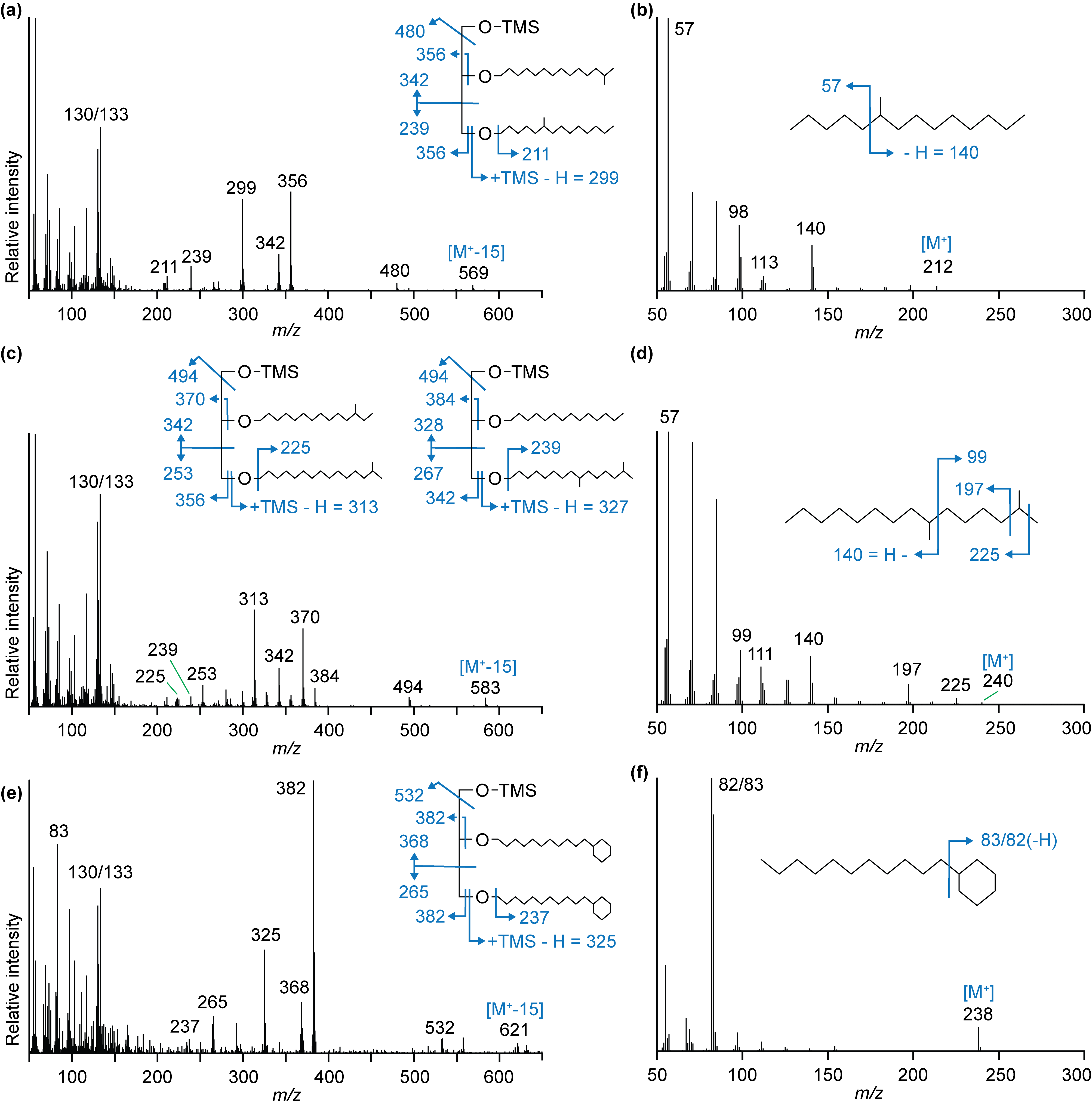


**Figure S2:** Mass spectra and structures of novel DAGEs and related compounds after ether cleavage. Arrows and numbers in blue show relevant mass fragmentation of the compounds. TMS = trimethylsilyl, [M+] = molecular ion. Compounds shown are (a) C_33_ (6me-C_14_/*iso*-C_15_) DAGE, (b) 6me-C_14_ alkane after ether cleavage, (c) C_34_ (*iso*-C_16_/*anteiso*-C_15_) DAGE co-eluting with C_34_ (dimethyl-C_15_/*n*-C_14_) DAGE, (d) 2,7-dimethyl-C_15_ alkane after ether cleavage, (e) C_37:2_ (cyclohexyl-C_11_/cyclohexyl-C_11_) DAGE and (f) cyclohexyl-C_11_ alkane after ether cleavage.
